# Defect‐Rich 2D Layered Double Hydroxides Enhance Sonodynamic Antibacterial Therapy

**DOI:** 10.1002/advs.202524216

**Published:** 2026-01-20

**Authors:** Qian Liu, Yu Yang, Rui Zhao, Linwei Huang, Xingyu Qi, Min Wu, Jianliang Shen

**Affiliations:** ^1^ Zhejiang Key Laboratory of Ophthalmic Drug Discovery and Medical Device Research Eye Hospital Wenzhou Medical University Wenzhou P. R. China; ^2^ Zhejiang Engineering Research Center for Tissue Repair Materials Wenzhou Institute University of Chinese Academy of Sciences Wenzhou Zhejiang P. R. China; ^3^ State Key Laboratory of Chemical Resource Engineering Beijing Advanced Innovation Center For Soft Matter Science and Engineering Beijing University of Chemical Technology Beijing P. R. China

**Keywords:** antibacterial, defect engineering, LDHs, SDT

## Abstract

Sonodynamic Therapy (SDT) is a non‐invasive therapeutic strategy for combating antibiotic‐resistant infections. However, current sonosensitizers typically show limited reactive oxygen species (ROS) output under ultrasound (US) irradiation. While layered double hydroxides (LDHs) feature tunable architectures and favorable biocompatibility for biomedical applications, their potential in SDT‐driven antibacterial systems remains underexplored. Here, we introduce a defect engineering paradigm based on a facile acid‐etching method to construct defect‐rich 2D DR‐ZnCuW‐LDH nanosheets. This ambient‐condition approach enables flexible defect‐phase engineering without energy‐intensive processing. Through this straightforward treatment, the nanosheets undergo a crystalline‐to‐polycrystalline phase transition, form abundant defects with oxygen vacancies (OVs), and narrow the bandgap (E*g*) from 3.29 to 1.80 eV, thereby markedly improving electron‐hole separation. Remarkably, DR‐ZnCuW‐LDH nanosheets exhibit a fourfold increase in ROS generation under US irradiation compared to pristine ZnCuW‐LDH, significantly enhancing its performance as an inorganic sonosensitizer for antibacterial applications. This substantial improvement stems from strategically introduced defects and the phase transformation‐induced electronic structure modification. Both in vitro and in vivo evaluations validate the exceptional antibacterial efficacy of DR‐ZnCuW‐LDH nanosheets under US, establishing a versatile platform for sonodynamic antibacterial applications.

## Graphical abstract

This study develops defect‐rich ultrathin ZnCuW‐LDH nanosheets (DR‐ZnCuW‐LDH) via acid etching. DR‐ZnCuW‐LDH exhibits fourfold enhanced US‐triggered ROS production due to oxygen defects and electronic modulation, demonstrating potent antibacterial efficacy in vitro/vivo. This defect engineering strategy presents an advanced inorganic sonosensitizer platform for SDT applications.

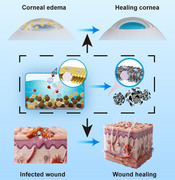

## 1 Introduction

Bacterial infections, especially those caused by multidrug‐resistant (MDR) and even pan‐drug‐resistant strains, have become a major global health threat, while the pipeline of effective new antibiotics remains extremely limited [1, 2, 3, 4]. Moreover, biofilm‐mediated persistent infections in deep tissues often resist conventional therapies due to poor deep‐seated infections are frequently biofilm‐mediated, characterized by poor drug penetration [5, 6]. Consequently, there is an urgent need for nontraditional antimicrobial modalities that bypass resistance mechanisms while ensuring biosafety. Reactive oxygen species (ROS)‐based strategies, as promising non‐antibiotic alternatives, induce oxidative damage to bacterial lipids, proteins, and DNA indiscriminately, rather than on a single molecular target [7]. This multi‐site damage mechanism enables ROS to efficiently eradicate drug‐resistant bacteria while greatly reducing the likelihood of inducing additional resistance compared with conventional antibiotics.

Sonodynamic therapy (SDT), initially developed for oncology, is increasingly recognized as a noninvasive approach for bacterial infections [8, 9, 10, 11, 12]. Although some sonosensitizers may lack active biological targeting ligands, the safety of SDT is inherently secured by the high spatial resolution of ultrasound (US). The generation of ROS is strictly confined to the localized region of US irradiation, ensuring that surrounding normal tissues remain unaffected even without specific molecular targeting. This physical confinement minimizes potential side effects, distinguishing SDT from systemic chemical treatments. Compared with light‐triggered therapeutic strategies such as photodynamic therapy (PDT) and photothermal therapy (PTT), SDT offers deeper tissue penetration, enabling targeted, noninvasive treatment of deep‐seated or widespread infections [13, 14]. Upon US activation, sonosensitizers generate ROS to eradicate bacteria. Unlike traditional antibiotics, SDT affords precise spatial targeting, minimal systemic side effects, and a lower propensity for resistance due to localized ROS production [9, 15, 16, 17]. Notably, ongoing clinical trials (e.g., NCT05362409, NCT05580328) are actively evaluating the therapeutic efficacy of SDT, either as a standalone treatment or in combination with other modalities [8, 18]. These studies underscore the growing momentum behind SDT as a clinically viable solution for complex infections, including those involving resistant bacteria. Although organic sonosensitizers, including curcumin, indocyanine green, phaeochromocytoma B, porphyrins, and their derivatives shown therapeutic potential, they often suffer from limited bioavailability, chemical stability, and rapid clearance [9, 19, 20, 21]. In contrast, inorganic sonosensitizers (e.g., TiO_2_、Cu_2_O, and MnOx) feature higher chemical and thermal stability and superior acoustic properties, making them attractive for SDT [9, 15, 21]. However, their intrinsically wide bandgaps (E*g*) severely limit their electron (e^−^)‐hole (h^+^) (e–h^+^) separation efficiency under US excitation, resulting in suboptimal ROS yields and restricted SDT performance [8, 22, 23]. For instance, TiO_2_produces relatively fewer ROS due to its large bandgap of 3.2 eV and the rapid e^−^‐h^+^ recombination [24]. Thus, designing new inorganic sonosensitizers with enhanced charge separation and narrower bandgaps is crucial for efficient ROS generation.

Reducing the bandgap of inorganic sonosensitizers, increasing their surface area, and enhancing the effective mass of e^−^‐ h^+^ can all contribute to improved charge separation and SDT efficiency. Strategies such as constructing heterogeneous structures, doping with other elements, and introducing oxygen vacancy (OVs) defects are widely used to suppress rapid charge recombination by capturing sonically activated electrons. Oxygen defect engineering has emerged as particularly effective, serving as local electron reservoirs that prolong carrier lifetimes and regulate interfacial redox dynamics [25, 26, 27, 28]. For example, Yang et al. introduced OVs into BiVO_4_ nanosheets, achieving the generation of hydroxyl radicals (·OH) and singlet oxygen (^1^O_2_) under US irradiation compared to their defect‐free counterparts [29]. Wang et al. demonstrated that PEG‐TiO_1+x_NRs with oxygen‐deficient structure can enhance SDT performance [30]. These findings validate the potential of defect engineering in improving SDT efficiency. Conventional methods for creating OVs (e.g., hydrogenation reduction and high‐temperature annealing) can cause structural damage, particle coarsening, compromise biocompatibility, and colloidal dispersity. In contrast, acid etching can introduce defects under mild conditions and has been leveraged to enhance photothermal‐/chemotherapy in LDH‐based systems [11, 31, 32]. Li et al. designed the defect‐rich multifunctional Cu‐doped layered double hydroxide (Cu‐LDH) nanoparticles by acid treatment to achieve non‐invasive imaging‐guided combination therapy [31]. Also, Hu et al. developed a defect‐rich sonosensitizer a‐CoW‐LDH for SDT by acid etching to achieve tumor eradication in vivo [11]. Yet, efficient inorganic sonosensitizers with optimized defect configurations for robust ROS generation in sonodynamic antibacterial therapy remain scarce.

2D Layered double hydroxides (LDHs) have attracted extensive interest owing to their unique lamellar architectures and tunable chemical compositions [10, 33]. LDHs are typically composed of divalent and trivalent metal ions arranged in brucite‐like layers and possess an interlayer region that can host various functional molecules or guest ions [22, 34]. LDHs have been demonstrated to be promising nanocarriers due to their excellent biocompatibility, acid‐responsive biodegradability, and diverse chemical compositions and structures [32, 35]. They are widely utilized in drug delivery, nanozymes, and sensors. For instance, LDHs can facilitate targeted transport and controlled release of antitumor drugs or gene molecules [11, 36, 37]. Additionally, functionalized LDHs have been investigated for their potential in detecting and catalyzing specific substances [10, 16, 32, 35]. However, the use of 2D LDHs as sonosensitizers for sonodynamic antibacterial therapy remains a conspicuous knowledge gap. Although some studies have examined the inherent antibacterial properties of LDHs, systematic research on enhancing their sonodynamic activation and ROS production capabilities is still lacking [38, 39, 40]. Therefore, developing LDHs‐based materials capable of efficiently generating ROS under US stimulation while maintaining high biosafety holds significant promise for expanding SDT in antibacterial therapy, particularly for bacterial infectious diseases.

**SCHEME 1:**
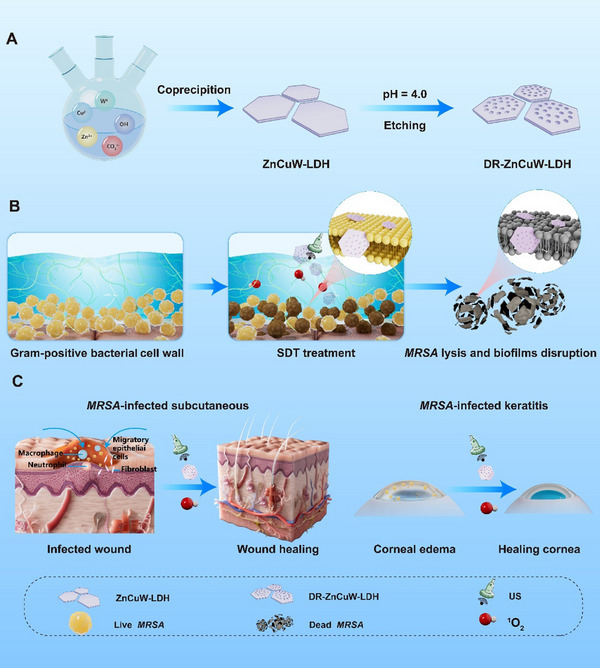
Schematic illustration of the preparation of DR‐ZnCuW‐LDH nanosheets and their antibacterial performance with Sonodynamic therapy. (A) Synthesis of ZnCuW‐LDH and DR‐ZnCuW‐LDH nanosheets. (B) DR‐ZnCuW‐LDH with US irradiation enhances antibacterial disruption and eradicates *MRSA* biofilm in vitro. (C) Therapeutic evaluation of DR‐ZnCuW‐LDH nanosheets in vivo.

Here, we report a defect‐engineering strategy based on mild acid etching to construct defect‐rich DR‐ZnCuW‐LDH nanosheets that undergo a crystalline‐to‐polycrystalline phase transition, form abundant Ovs, reduce the bandgap Eg, and thereby promote e^−^‐ h^+^ separation. Under US irradiation, DR‐ZnCuW‐LDH nanosheets exhibited superior ^1^O_2_ generation activity with an activity approximately 4 times greater than that of the original single‐crystal ZnCuW‐LDH nanosheets. Both in vitro and in vivo studies showed that DR‐ZnCuW‐LDH nanosheets effectively triggered bacterial apoptosis and eradicated infections. This study highlights the critical role of the defect structure of 2D LDH nanosheets in determining their efficacy in sonodynamic antibacterial therapy. Through systematic structure‐activity relationship studies, we conclusively establish that OVs defect engineering in DR‐ZnCuW‐LDH expands the antimicrobial toolkit for LDH‐based nanomaterials and pioneers a US‐responsive therapeutic platform capable of eradicating drug‐resistant biofilms with millimeter precision (Scheme 1).

## 2 Results and Discussion

### 2.1 Preparation and Characterization of ZnCuW‐LDH

**FIGURE 1:**
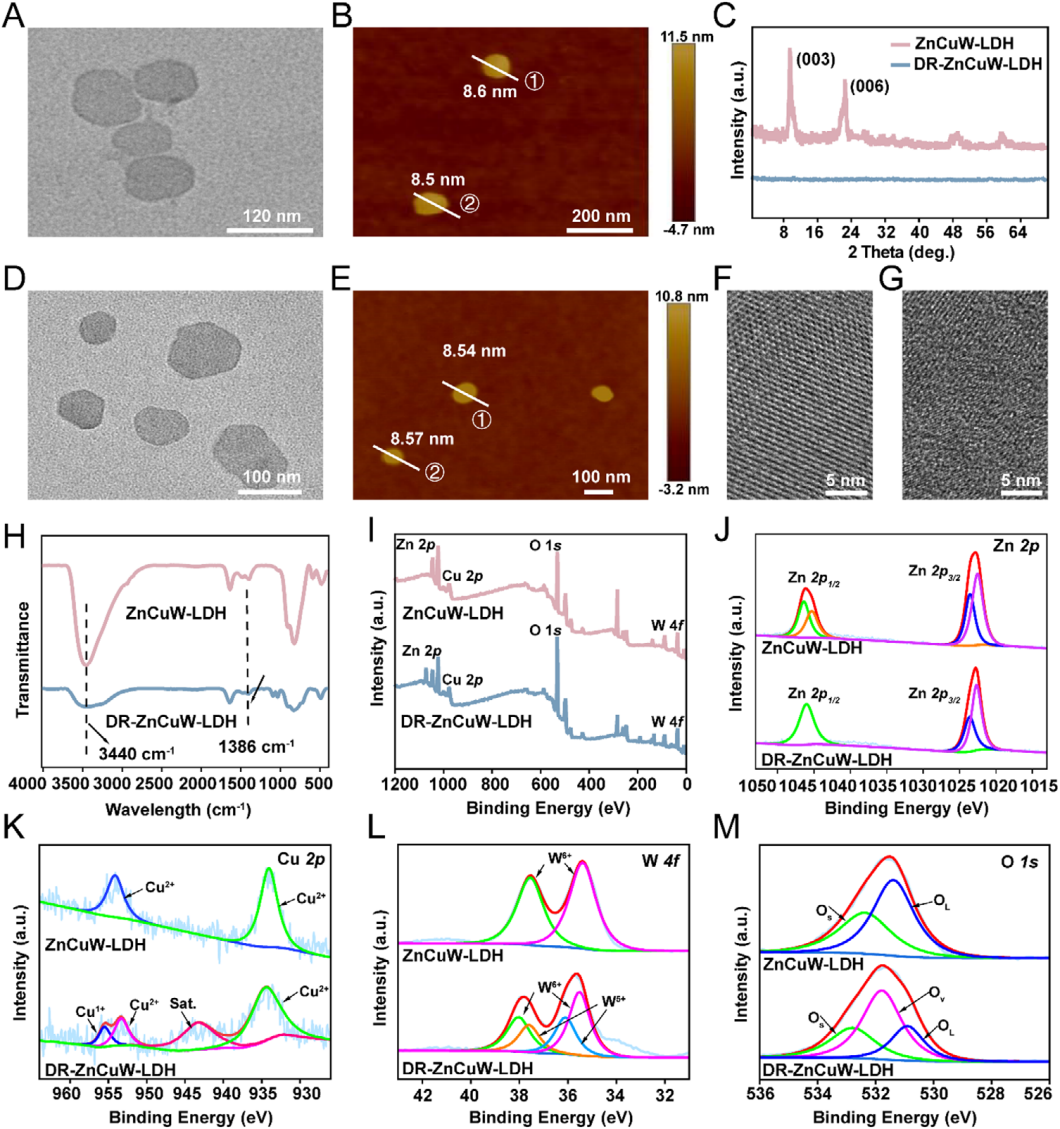
Structural characterization of ZnCuW‐LDH and DR‐ZnCuW‐LDH nanosheets. (A) Representative TEM image of ZnCuW‐LDH nanosheets. (B) AFM images of ZnCuW‐LDH nanosheets. (C) XRD patterns of ZnCuW‐LDH and DR‐ZnCuW‐LDH nanosheets. (D) TEM images and (E) AFM images of DR‐ZnCuW‐LDH. Atomic‐resolution STEM images of typical (F) ZnCuW‐LDH and (G) DR‐ZnCuW‐LDH nanosheets. (H) FTIR spectra of ZnCuW‐LDH and DR‐ZnCuW‐LDH nanosheets. (I) XPS spectra for ZnCuW‐LDH and DR‐ZnCuW‐LDH nanosheets. (J‐M) Zn *2p*, Cu *2p*, W *4f*, and O *1s* XPS spectra of ZnCuW‐LDH and DR‐ZnCuW‐LDH nanosheets.

Ultrathin 2D nanosheets ZnCuW‐LDH were synthesized via a low‐temperature hydrothermal method as previously reported [11, 32]. Transmission electron microscope (TEM) images indicated that the ZnCuW‐LDH nanosheet displays plate‐like morphology (Figure 1A). The dimensions of the ZnCuW‐LDH nanosheets are approximately 100–105 nm, and the energy‐dispersive X‐ray (EDX) elemental mapping revealed a uniform distribution of Zn, Cu, and W across individual nanosheets (Figure S1A,B). Atomic force microscopy (AFM) height profiles further highlighted the ultrathin characteristics, indicating a thickness of roughly 8.0 ‐ 9.0 nm (Figure 1B; Figure S2). X‐ray diffraction (XRD) patterns of ZnCuW‐LDH displayed characteristic diffraction peaks at 2θ = 11.52° and 24.12°, indexed to the (003) and (006) planes, confirming the layered structure and good crystallinity (Figure 1C). Furthermore, the crystalline structure of ZnCuW‐LDH nanosheets can be transformed into DR‐ZnCuW‐LDH nanosheets through an acid etching treatment. The XRD pattern of DR‐ZnCuW‐LDH showed that the typical diffraction peaks disappeared (Figure 1C). Both ZnCuW‐LDH and DR‐ZnCuW‐LDH nanosheets have good stability for at least 7 days (Figure S3). To further validate dispersion robustness under biologically relevant conditions, we have monitored the hydrodynamic diameter of DR‐ZnCuW‐LDH nanosheet in PBS, DMEM, and water by DLS every two days for 2 weeks. No appreciable changes in size distributions were observed in any medium throughout the test window, indicating excellent colloidal stability in simulated physiological fluids (Figure S4). As shown in Figure 1D, the TEM images of DR‐ZnCuW‐LDH nanosheets demonstrate that they preserve the 2D structure of ZnCuW‐LDH nanosheets, albeit with a slightly reduced size of 80–100 nm. Additionally, the AFM height profile of DR‐ZnCuW‐LDH nanosheets has remained with negligible changes with ZnCuW‐LDH nanosheets in Figure 1E and presented an ultrathin morphology with a thickness of approximately 8.0 ‐ 9.0 nm in Figure S5. Furthermore, the STEM image indicates that the ZnCuW‐LDH nanosheets display a single‐crystalline structure. In contrast, the STEM image of the DR‐ZnCuW‐LDH nanosheet (Figure 1F) indicates no obvious crystalline lattice fringes, proving its amorphous structure. In comparison, the atomic‐resolution STEM image of a typical DR‐ZnCuW‐LDH nanosheet (Figure 1G) shows no distinct crystalline‐lattice fringes, which further confirms its polycrystalline nature.

### 2.2 Characterization of DR‐ZnCuW‐LDH

Fourier transform infrared (FT‐IR) spectroscopy was utilized to investigate the structural properties and functional groups present in the ZnCuW‐LDH and DR‐ZnCuW‐LDH nanosheets. As shown in Figure 1H, the emergence of absorption bands at 3400 and 1386 cm^−1^, which are attributed to the stretching vibrations of hydroxyl groups (O‐H) and C═O (CO_3_^2−^), demonstrates the successful synthesis of ZnCuW‐LDH. Furthermore, acid etching has negligible performance on the LDH structure. To further investigate the elemental composition and chemical states of ZnCuW‐LDH and DR‐ZnCuW‐LDH nanosheets, X‐ray photoelectron spectroscopy (XPS) was conducted. In Figure 1I, peaks corresponding to Zn, Cu, W, and O are observed in the survey spectra of both ZnCuW‐LDH and DR‐ZnCuW‐LDH nanosheets. In the Zn *2p* XPS spectra (Figure 1J), peaks at 726.38 and 712.88 eV correspond to Zn^2+^ 2*p*_3/2_ and 2*p*_1/2_, suggesting the existence of Zn^2+^ in ZnCuW‐LDH nanosheets. The binding energies remain largely unchanged before and after etching, as Zn predominantly exists in the divalent state (Zn^2+^) (Figure 1J). This indicates that the etching process has a minimal impact on the Zn element. In the Cu 2*p* XPS spectrum of ZnCuW‐LDH, the peaks at 934.0 and 954.1 eV are assigned to Cu^2+^. In contrast, the Cu 2*p* spectra of DR‐ZnCuW‐LDH show new binding peaks at 953 eV can be assigned to the Cu^+^, manifesting the partial reduction of Cu^2+^ to Cu^+^. This reduction occurs because oxygen vacancies, which result from the loss of oxygen atoms, effectively lower the oxidation state of high‐valence metal ions in the nanosheet layers during acid etching (Figure 1K). The high‐resolution XPS W 4*f* spectrum of the ZnCuW‐LDH nanosheets reveals two peaks at 35.41 and 37.57 eV, corresponding to W4*f*_5/2_ and W4*f*_7/2_, respectively. In contrast to the ZnCuW‐LDH nanosheets, the DR‐ZnCuW‐LDH nanosheets exhibit two additional peaks at 37.64 and 35.54 eV, which are associated with W^5+^ 4*f*_5/2_ and W^5+^ 4*f*_7/2_, indicating a partial reduction of hexavalent tungsten (W^6+^) to pentavalent tungsten (W^5+^) during the acid etching in Figure 1L. These results demonstrate that the etching process effectively induces the formation of oxygen vacancies (OVs) and alters the valence states of the Cu and W elements.

Furthermore, X‐ray photoelectron spectroscopy (XPS) analysis of the O *1s* region (Figure 1M) further confirms the presence of different oxygen species in the ZnCuW‐LDH and DR‐ZnCuW‐LDH nanosheets. Specifically, binding energies at 530.78 and 532.91 eV correspond to lattice oxygen (OL) and adsorbed oxygen (OS) in the ZnCuW‐LDH nanosheets. However, compared to the ZnCuW‐LDH nanosheets, a new peak at 531.71 eV emerges in the DR‐ZnCuW‐LDH nanosheets, which is attributed to the formation of abundant oxygen vacancies (OVs). Thus, it can be inferred that the acid etching process is an effective, straightforward method for facilitating the transformation of 2D ZnCuW‐LDH nanosheets from a crystalline to a defect phase. Additionally, the ESR spectra indicate that the DR‐ZnCuW‐LDH nanosheets display a more intense signal at G = 2.2 than the ZnCuW‐LDH nanosheets, suggesting that acid etching introduces a significant number of defects (Figure S6). This observation is consistent with previous studies on defect engineering [32].

### 2.3 SDT Performance of DR‐ZnCuW‐LDH Nanosheets

**FIGURE 2:**
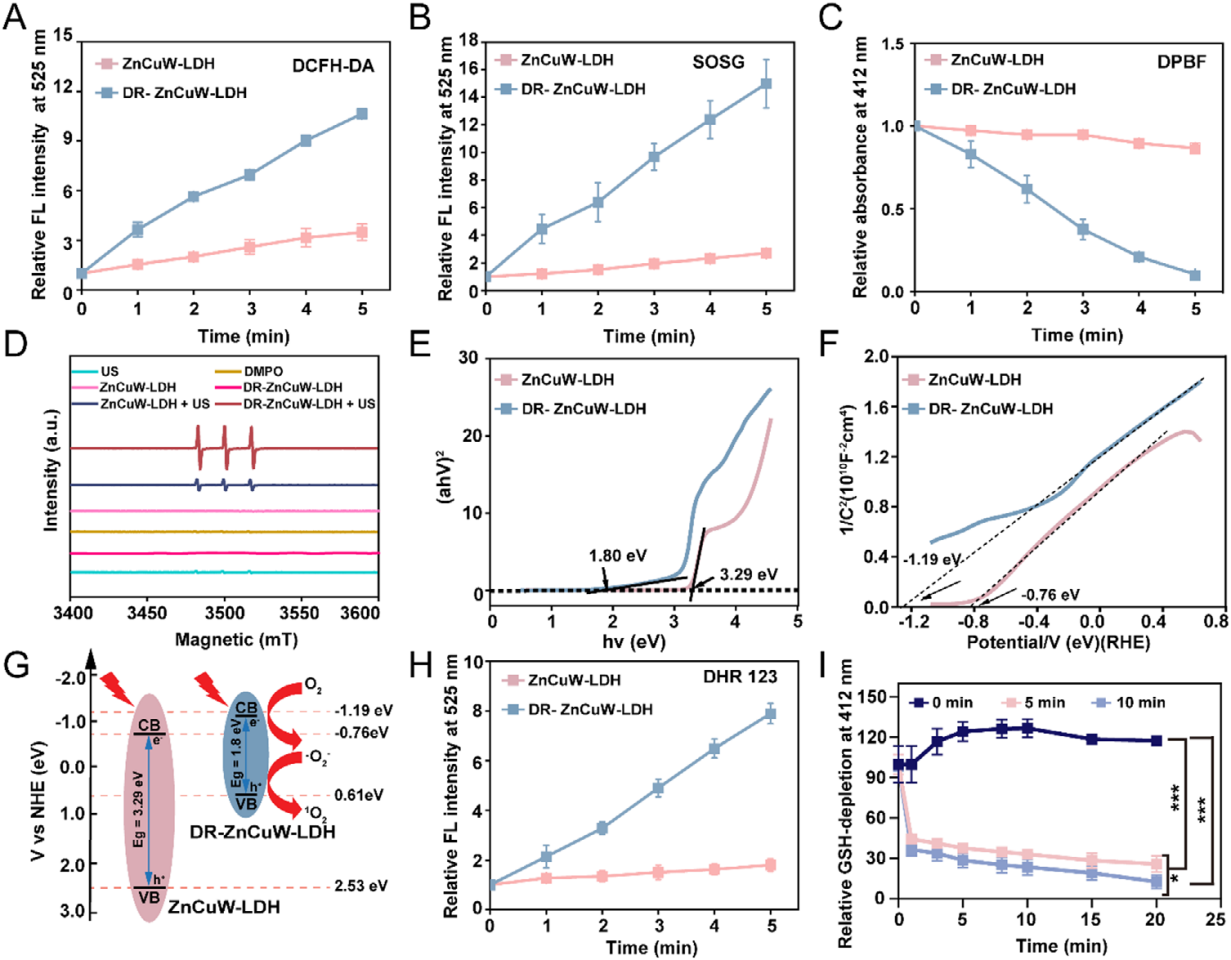
^1^O_2_ generation of DR‐ZnCuW‐LDH nanosheets under US stimulation. (A) The relative fluorescence intensity of DCFH‐DA under US irradiation (1 MHz, 1.0 W cm^−2^, 5 min) of ZnCuW‐LDH and DR‐ZnCuW‐LDH nanosheets. (B) Relative fluorescence intensity of ^1^O_2_ probe SOSG in the presence of ZnCuW‐LDH and DR‐ZnCuW‐LDH nanosheets under US irradiation (1.0 MHz, 1.0 W cm^−2^, 5 min). (C) The relative absorption changes of DPBF at 412 nm between ZnCuW‐LDH and DR–ZnCuW‐LDH nanosheets under US irradiation (1.0 MHz, 1.0 W cm^−2^, 5 min). (D) ESR spectra of ^1^O_2_ for ZnCuW‐LDH and DR‐ZnCuW‐LDH nanosheets. (E) Bandgap energy, (F) Mott‐Schottky plots, and (G) band diagrams of ZnCuW‐LDH and DR‐ZnCuW‐LDH nanosheets. (H) Relative fluorescence intensity changes of DHR 123 FL intensity in the presence of ZnCuW‐LDH and DR‐ZnCuW‐LDH under US irradiation. (I) GSH‐depletion of DR‐ZnCuW‐LDH nanosheets under US irradiation at different times. The ZnCuW‐LDH solution and DR‐ZnCuW‐LDH solution were prepared at a concentration of 50 µg mL^−1^. Data are presented as mean ± s.d. (n =3), ^*^ *p* < 0.05, ^**^ *p* < 0.01, and ^***^ *p* < 0.001.

To investigate the defect engineering on the SDT performance of DR‐ZnCuW‐LDH nanosheets. The sonodynamic performance of DR‐ZnCuW‐LDH nanosheets sonosensitizers was systematically investigated. To optimize the etching conditions, the singlet oxygen sensor green (SOSG) fluorescent probe was employed as a ROS capturer. Initially, we conducted a thorough investigation into how different conditions‐such as pH levels (4.0, 5.0, 6.0, and 7.0), etching durations (2, 4, 8, 12, and 24 h), and Cu doping ratios (3%, 5%, 10%, and 20%)‐affect the ROS generation capacity of DR‐ZnCuW‐LDH nanosheets. As illustrated in Figures S7 and S8, a gradual increase in the fluorescence intensity of SOSG was observed for nanosheets etched at pH 4.0 under US irradiation (1 MHz, 1.0 W cm^−2^, 5 min). Notably, ZnCuW‐LDH nanosheets etched for 4 h demonstrated the highest fluorescence intensity under pH 4.0 conditions. Subsequently, we evaluated the ROS generation performance of DR‐ZnCuW‐LDH nanosheets with varying Cu doping ratios (3%, 5%, 10%, and 20%) (Figure S9). The results revealed that DR‐ZnCuW‐LDH nanosheets doped by 5% Cu and exhibited the most effective singlet oxygen (^1^O_2_) generation capacity upon US irradiation (1 MHz, 1.0 W cm^−2^, 5 min). Based on these findings, we selected the etching condition of pH 4.0 for 4 h with 5% Cu doping in ZnCuW‐LDH nanosheets for subsequent experiments. As shown in Figure 2A, the DR‐ZnCuW‐LDH nanosheets exhibited stronger DCFH‐DA fluorescence intensity, which exhibits an activity approximately 3.7 times greater than that of the ZnCuW‐LDH nanosheets under US irradiation (1 MHz, 1.0 W cm^−2^, 5 min).

The generation of ^1^O_2_ of DR‐ZnCuW‐LDH nanosheets was also monitored using SOSG and 1,3‐diphenylisobenzofuran (DPBF), a well‐established indicator of ^1^O_2_. As shown in Figure 2B, the DR‐ZnCuW‐LDH nanosheets demonstrated stronger ROS generation performance than ZnCuW‐LDH nanosheets by about 4 times. Additionally, the UV–vis‐near‐infrared (UV–vis‐NIR) absorption spectrum (300–480 nm) of DPBF, together with DR‐ZnCuW‐LDH nanosheets, showed a rapid decline with prolonged US irradiation (Figure 2C). In contrast, the DPBF absorption spectrum in the presence of ZnCuW‐LDH nanosheets exhibited only minor changes under US irradiation, indicating the strong capability of DR‐ZnCuW‐LDH toward ^1^O_2_ generation (Figure 2C). Furthermore, electron spin resonance (ESR) spectroscopy was utilized to monitor the production of singlet oxygen (^1^O_2_) through the use of 2,2,6,6‐tetramethyl‐4‐piperidone (TEMP) [41]. Following exposure to ultrasonic irradiation (1 MHz, 1.0 W cm^−2^, 5 min), a distinct 1:1:1 signal characteristic of ^1^O_2_ was observed in DR‐ZnCuW‐LDH group under US irradiation (1.0 MHz, 1.0 W cm^−2^, 5 min) (Figure 2D) Additionally, no 1:2:2:1 signal corresponding to hydroxyl radicals (·OH) was detected, indicating no other reactive oxygen species are generated (Figure S10).

**FIGURE 3:**
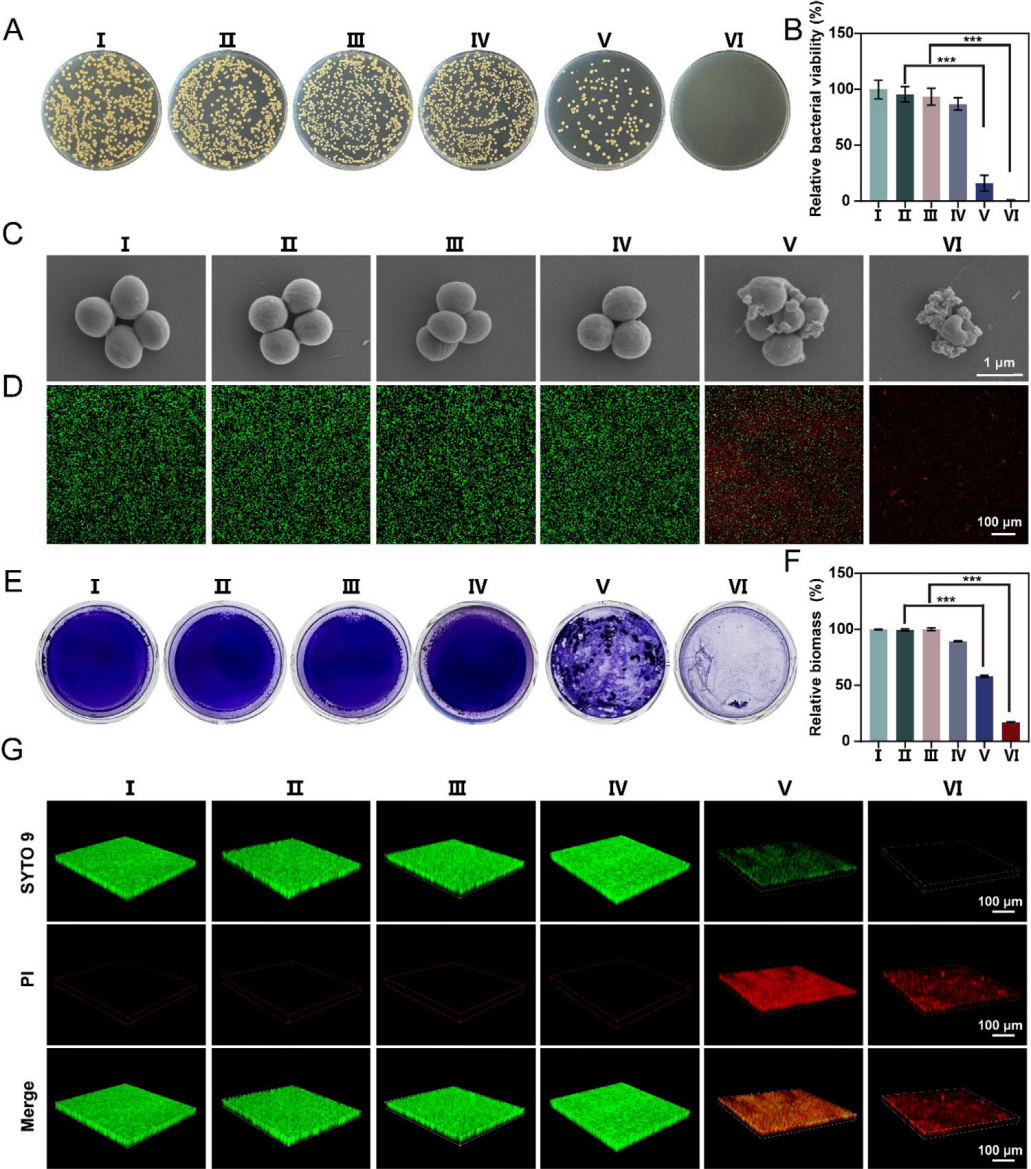
Antibacterial and anti‐biofilm performance of DR‐ZnCuW‐LDH nanosheets in vitro. (A) Representative images of agar plates and (B) the relative bacterial viability quantified from colony counts after the indicated treatments. PBS (I), PBS +US 5 min (II), PBS +US 10 min (III), DR‐ZnCuW‐LDH nanosheets (IV), DR‐ZnCuW‐LDH nanosheets + US 5 min (V), and DR‐ZnCuW‐LDH nanosheets + US 10 min (VI). (C) SEM images of *MRSA* with the given treatments. (D) Live/dead fluorescence staining of *MRSA* treated with different groups. The green fluorescence (SYTO9) and the red fluorescence (Propidium, PI). (E) Crystal violet staining of *MRSA* biofilms formed under different treatments and (F) corresponding quantitative analysis of biofilm biomass. (G) CLSM images of *MRSA* biofilms after the indicated treatments. The DR‐ZnCuW‐LDH solution was prepared at a concentration of 50 µg mL^−1^. Data are presented as mean ± s.d. (n =3), ^*^ *p* < 0.05, ^**^ *p* < 0.01, and ^***^ *p* < 0.001.

To gain deeper insight into the mechanism of ROS generation mediated by the US, the band structures of ZnCuW‐LDH and DR‐ZnCuW‐LDH nanosheets were examined using UV–vis‐NIR spectroscopy. As shown in Figure 2E, the bandgaps (E*g*) of the ZnCuW‐LDH and DR‐ZnCuW‐LDH nanosheets were found to be 3.29 and 1.8 eV, respectively. To determine their conduction band (CB) positions, Mott‐Schottky plots were generated to reveal CB potentials of −1.19 eV for DR‐ZnCuW‐LDH and −0.76 eV for ZnCuW‐LDH, as illustrated in Figure 2F. Consequently, the associated valence band (VB) potentials were determined to be 2.53 eV for ZnCuW‐LDH and 0.61 eV for DR‐ZnCuW‐LDH nanosheets, respectively. Based on these findings, the energy band structures of ZnCuW‐LDH and DR‐ZnCuW‐LDH nanosheets are clearly illustrated in Figure 3G. DR‐ZnCuW‐LDH nanosheets enable efficient excitation and separation of e^−^‐h^+^ pairs under US irradiation. In contrast to ZnCuW‐LDH nanosheets, DR‐ZnCuW‐LDH nanosheets exhibit a lower E*g*, making them more excited and facilitating the separation of charge carriers, with h^+^ occupying the VB and e^−^ residing in the CB. The e^−^ and h^+^ are situated in CB and VB, with the electrons generated by ultrasound being discharged into the surrounding environment, where they react with O_2_ to form the intermediate species ·O_2_^−^. Subsequently, ·O_2_^−^ combines with h^+^ to produce the final product, ^1^O_2_. The generation of ·O_2_^−^ was confirmed using the dihydrorhodamine 123 (DHR 123) probe, which emits a fluorescence signal at 525 nm upon reaction with ·O_2_^−^. In Figure 2H, a faint fluorescence signal from DHR 123 was detected in the presence of ZnCuW‐LDH nanosheets when subjected to ultrasound irradiation. (1.0MHz, 1.0 W cm^−2^, 5 min), while a robust fluorescence signal was observed in the presence of DR‐ZnCuW‐LDH nanosheets, suggesting an enhanced efficiency in the separation of e^−^ and h^+^ for these nanosheets.

Glutathione (GSH) depletion profoundly influences the pathophysiology of infected tissue healing by intensifying oxidative stress, perpetuating inflammatory dysregulation, and impairing cellular processes essential for tissue repair. Infected areas are typically characterized by elevated levels of ROS and reactive nitrogen species (RNS), derived both from immune cell responses and microbial metabolism, which disrupt redox homeostasis when GSH concentrations fall below critical thresholds [42, 43]. The resultant oxidative damage‐ including lipid peroxidation, protein oxidation, and DNA lesions‐ attenuates the proliferation of fibroblasts and migration of keratinocytes, thus hindering extracellular matrix remodeling and re‐epithelialization [44, 45]. Notably, recent evidence implicates GSH depletion in triggering ferroptosis, an iron‐dependent form of programmed cell death that aggravates tissue damage and impedes structural and functional recovery of wounded areas [46, 47]. The absorption peak of the DTNB (5,5'‐Dithiobis (2‐nitrobenzoic acid)) probe at 412 nm was applied to reflect GSH depletion. After US stimulation for 5 min or 10 min, DR‐ZnCuW‐LDH nanosheets can efficiently decrease the absorption of DTNB at once and continuously decrease over time (Figure 2I). According to statistical data, US treatment of DR‐ZnCuW‐LDH nanosheets reduces GSH consumption to around 10 min and exhibits a clear time‐dependent effect in Figure 2I. Hence, DR‐ZnCuW‐LDH nanosheets may represent a promising therapeutic avenue to ameliorate infection‐induced tissue destruction and promote efficient wound closure.

### 2.4 Antibacterial and Anti‐Biofilm Performance of LDH Nanosheets In Vitro

Motivated by the excellent ROS generation capability and sonodynamic properties under US stimulation of DR‐ZnCuW‐LDH nanosheets, we further evaluated the potential antibacterial activity of DR‐ZnCuW‐LDH nanosheets. To evaluate the antibacterial efficacy of DR‐ZnCuW‐LDH nanosheets activated by US irradiation, a series of experiments was performed to evaluate bacterial viability, biofilm disruption, and structural changes in bacterial cells. The most representative Gram‐positive strain, *MRSA*, and Gram‐negative strain, *P. aeruginosa*, were selected as model bacteria for subsequent investigations. The biocompatibility of materials is a critical factor for their potential biological applications. To examine this, bacterial survival was tested after co‐culturing with DR‐ZnCuW‐LDH nanosheets at varying concentrations. The results indicated that, after two hours of co‐culture at different material concentrations, followed by analysis using the dilution plating method, the nanosheets at a concentration of 100 µg mL^−1^ did not exhibit significant toxic side effects on *MRAS* and *P. aeruginosa*. Even at a concentration of 200 µg mL^−1^, the bacterial survival rate remained at 80%, as shown in Figures S11A and S12A. Additionally, bacterial growth curves were plotted by measuring the optical density (OD = 600) at different time points, further confirming that DR‐ZnCuW‐LDH nanosheets exhibit good biocompatibility with *MRAS* and *P. aeruginosa* in Figures S11B,C and S12B,C. Under US stimulation, the DR‐ZnCuW‐LDH nanosheets solution (50 µg mL^−1^) can release ^1^O_2_, which could combat bacteria to achieve an antibacterial effect. Following US (1.0 MHz, 1.0 W cm^−2^) for 5 min, bacterial colonies significantly decreased to 16.09% and 23.4% for *MRSA* and *P. aeruginosa*, respectively (Figure 3A,B; Figure S13A,B). Notably, the DR‐ZnCuW‐LDH + US group exhibited exceptional sterilization efficacy, achieving nearly 100% bacterial elimination for both *P. aeruginosa* and *MRSA* under the treatment of US and laser irradiation for 10 min in Figure 3A,B and Figure S13A,B.

Additionally, the morphological changes of *MRSA* were examined by using scanning electron microscopy (SEM) (Figure 3C; Figure S13C). The PBS (I), PBS + US 5 min (II), and PBS + US 10 min (III) maintained an intact spherical morphology, and the cell membranes appeared smooth and intact, with no visible damage observed even after the US. However, varying degrees of membrane wrinkling and structural disruption were evident on the surface of *MRSA* treated with DR‐ZnCuW‐LDH solution (50 µg mL^−1^) following US irradiation, with the most pronounced damage. These findings further confirm the superior sonodynamic antibacterial properties of DR‐ZnCuW‐LDH nanosheets. To further confirm bacterial viability, live/dead staining was performed using SYTO‐9 and propidium iodide (PI). As shown in Figure 3D and Figure S13D, the PBS (I), PBS + US 5 min (II), and PBS + US 10 min (III) groups displayed predominantly green fluorescence, indicating live bacteria. In contrast, the DR‐ZnCuW‐LDH + US 5 min (V) and ZnCuW‐LDH + US 10 min (VI) exhibited a significant increase in red fluorescence, indicating bacterial membrane damage and cell death. These findings are consistent with the SEM results and further validate the antibacterial efficacy of the treatment.

Bacterial biofilms have contributed to over 60% of human infectious diseases and play a vital role in bacterial adhesion and growth [48, 49]. *MRSA* biofilms are complex formations made up of extracellular polymers such as polysaccharides, proteins, and nucleic acids. These structures protect bacteria from the host's immune responses and hinder the effective infiltration of antimicrobial agents. Moreover, biofilms can prevent the penetration of antibiotics and then decrease their antibacterial activities owing to the protection of extracellular polymeric substances (EPS) [49, 50]. The ability of DR‐ZnCuW‐LDH nanosheets to disrupt biofilms was assessed using crystal violet staining. As shown in Figure 3E and Figure S13E, biofilm biomass was significantly reduced in the DR‐ZnCuW‐LDH + US group (VI) compared to the control groups. Compared to PBS + US (II), an approximately 1.82‐fold and 2.47‐fold decrease for *MRSA* and *P. aeruginosa*, respectively, in the amount of biofilm biomass under US stimulation for 5 min (1 MHz, 1.0 W cm^−2^) in the DR‐ZnCuW‐LDH + US group (V) (Figure 3F; Figure S13F). Moreover, the DR‐ZnCuW‐LDH + US group (VI) could further enhance biofilm disruptions with 10 min US irradiation. Quantitative analysis (Figure 3F; Figure S12F) revealed that the DR‐ZnCuW‐LDH + US group achieved a biofilm biomass reduction of over 90% (*p* < 0.001), highlighting its superior biofilm‐disrupting capability. Confocal laser scanning microscopy (CLSM) was used to visualize biofilm structure and bacterial viability in three dimensions. As shown in Figure 3G and Figure S13G, the PBS (I), PBS + US 5 min (II), and PBS + US 10 min (III) groups exhibited dense and uniform biofilm structures with predominantly live bacteria (green fluorescence). In contrast, the DR‐ZnCuW‐LDH + US group (VI) displayed a disrupted biofilm structure with a significant increase and notable holes in dead bacteria (red fluorescence). The merged images further confirm the effective biofilm disruption and bacterial killing achieved by the treatment. DR‐ZnCuW‐LDH nanosheets can release ROS under US stimulation, which can be used to chemically degrade the EPS of biofilms and facilitate antibacterial activities [17, 50]. The results demonstrate that DR‐ZnCuW‐LDH nanosheets, when activated by US, exhibit potent antibacterial and biofilm‐disrupting effects. The synergistic interaction between DR‐ZnCuW‐LDH nanosheets and ultrasound leads to significant bacterial cell damage, biofilm disruption, and a marked reduction in bacterial viability. These findings highlight the potential of DR‐ZnCuW‐LDH nanosheets as a novel and effective strategy for combating bacterial infections and biofilm‐associated diseases.

To delineate how DR‐ZnCuW‐LDH nanosheets triggered ROS translate into bacterial injury under US, we quantified intracellular oxidant burden and membrane function in *MRSA* and *P. aeruginosa* under identical conditions. Confocal imaging with DCFH‐DA revealed a significant increase in intracellular ROS in the DR‐ZnCuW‐LDH + US groups compared to PBS, US alone, or nanosheets alone, with a clear exposure‐time dependence from 5 to 10 min (Figures S14 and S15). Concordantly, 3,3’‐dipropylthiadicarbocyanine Iodide (DiSC_3_(5)) assays were employed to evaluate bacterial membrane potential changes after various treatments. Lipophilic DiSC_3_(5) aggregated within the phospholipid bilayer of bacterial membranes, causing self‐quenching of dyes in PBS and single‐treatment controls. Nevertheless, treatments of DR‐ZnCuW‐LDH + US groups depolarized the bacterial membranes, disrupted the membrane potential, resulting in the release of DiSC_3_(5) and enhanced fluorescence in the supernatant (Figures S16A and S17A). Moreover, o‐nitrophenyl β‐D‐galactopyranoside (ONPG) hydrolysis revealed increased membrane permeability following DR‐ZnCuW‐LDH + US, indicative of compromised envelope barriers (Figures S16B and S17B). As shown in Figure 3C and Figure S13C, the original morphology of the bacteria in the DR‐ZnCuW‐LDH + US 10 min group was severely disrupted, exhibiting obvious shrinking and wrinkles in the membrane, resulting in the release of the bacterial contents. The bacterial protein leakage assays were conducted using a BCA protein assay kit following different treatments to further assess loss of membrane integrity (Figures S16C and S17C). Taken together, these results establish a cascade whereby US‐activated DR‐ZnCuW‐LDH nanosheets elevate intracellular oxidative stress, collapse the membrane potential, increase permeability, induce leakage of intracellular contents, and cause direct bacterial death.

### 2.5 Anti‐Infective Therapy of LDH Nanosheets in Wound Healing

Driven by the promising results from the in vitro antibacterial assays, further investigations were conducted to evaluate the therapeutic potential of DR‐ZnCuW‐LDH nanosheets. The cytotoxic effects of DR‐ZnCuW‐LDH nanosheets were evaluated using the CCK‐8 assay on L929 and HCEC cell lines. As shown in Figure S18, the DR‐ZnCuW‐LDH nanosheets exhibited minimal cytotoxicity. Even at a high concentration (150 µg mL^−1^) after 24 h of co‐incubation, cell viability remained above 85%. Considering their excellent ROS generation capabilities and sonodynamic properties, DR‐ZnCuW‐LDH nanosheets demonstrate significant potential as an efficient nanomaterial for sonodynamic antibacterial therapy in vivo.

**FIGURE 4:**
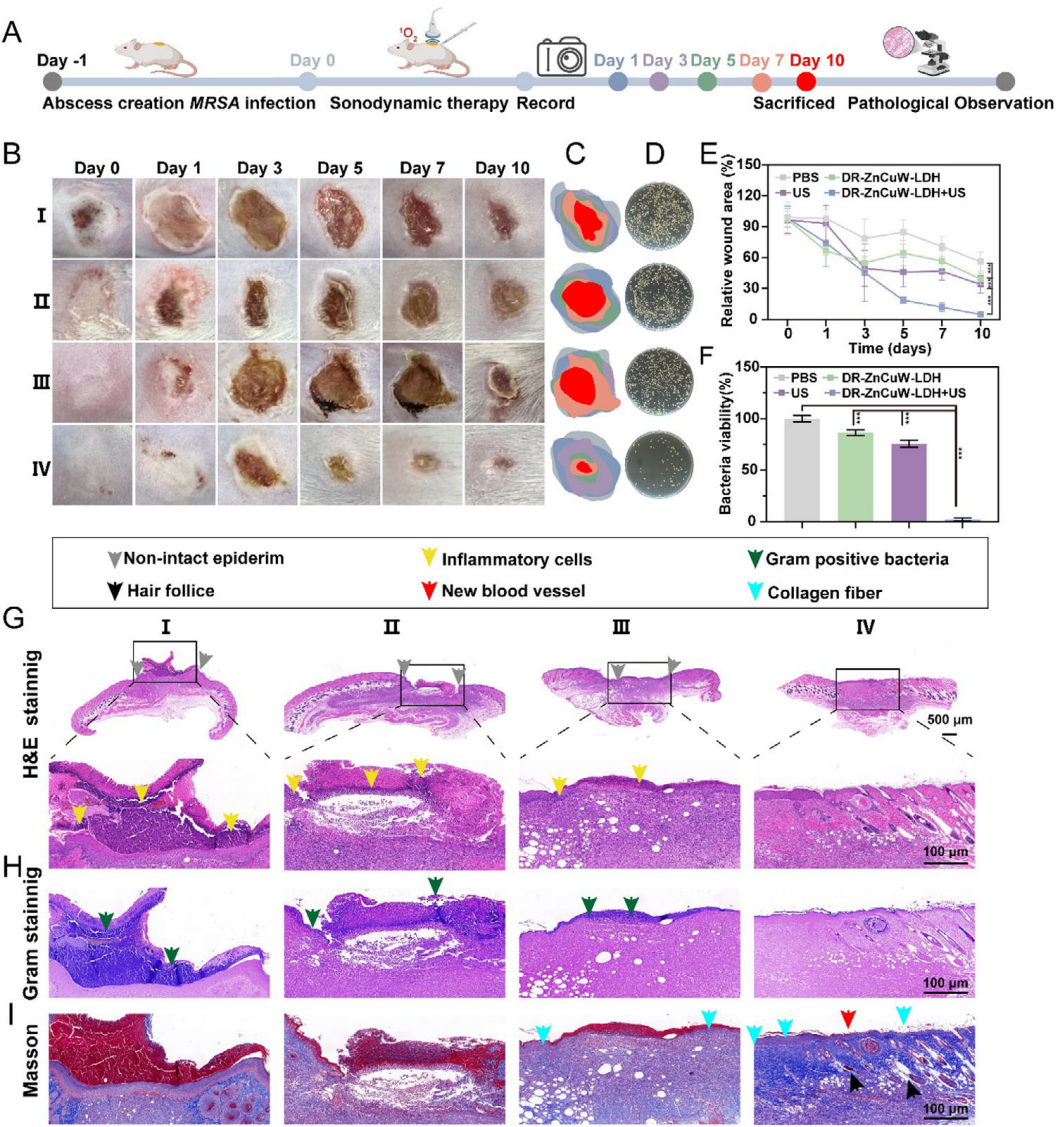
The therapeutic evaluation of DR‐ZnCuW‐LDH nanosheets on infected abscesses with *MRSA* in Wound Healing. (A) Schematic of the in vivo *MRSA* abscess model and sonodynamic therapy timeline, highlighting key observation time points. (B) Representative wound images of four treatment groups (I‐IV) at days 0, 1, 3, 5, 7, and 10. (C) The corresponding healing progress of abscesses for each treatment group in vivo. Gray, blue, purple, green, orange, and red represented abscess sizes on days 0, 1, 3, 5,7, and 10, respectively. (D) Images of bacterial colonies isolated from infected mouse abscesses across distinct treatment groups on day 3. (E) The ratio of abscess healing in mice from day 0 to day 10. (F) Corresponding bacterial viability of (D). (G) H&E, (H) Gram staining, and (I) Masson staining of tissue samples collected from abscess regions across the treatment groups on day 10. Data are expressed as mean ± SD (n = 3), with significance levels indicated as follows: ^*^ *p* < 0.05, ^**^ *p* < 0.01, and ^***^ *p* < 0.001.

To validate the SDT efficacy in clinically relevant scenarios involving tissue barriers, we deliberately selected a subcutaneous abscess model and a bacterial keratitis model that mimic deep‐seated or complex infections, which involve multi‐layered tissue structures that mimic complex infections. A murine subcutaneous abscess model, induced through local injection of *MRSA*, was employed to evaluate SDT efficacy and wound repair (Figure 4A). The mice were categorized into 4 distinct groups: PBS (I), DR‐ZnCuW‐LDH (II), US (III), and DR‐ZnCuW‐LDH+US (IV). Photographs of *MRSA*‐infected abscesses over a 10‐day treatment period are shown in Figure 4B. Mice treated with DR‐ZnCuW‐LDH+US (IV) exhibited the most significant recovery, with a gradual reduction in scar size over time (Figure 4C). By day 10, no significant improvement was observed in the PBS (I) group, which showed persistent secretion and purulent fluid leakage, indicating severe wound infection. The DR‐ZnCuW‐LDH (II) and US (III) groups displayed moderate wound healing with residual scarring (Figure 4C). Notably, the DR‐ZnCuW‐LDH+US (IV) group achieved the fastest recovery, with complete scar resolution by Day 10, attributed to the ROS generation facilitated by sonodynamic therapy (Figure 4B,E), underscoring its therapeutic potential. In addition, no obvious change in body weight over time indicates treatments in Figure S19.

The antibacterial performance of DR‐ZnCuW‐LDH+US was further evaluated by quantifying bacterial colony‐forming units (CFUs) on agar plates using tissue extracted from abscesses on day 3 (Figure 4D). Minimal bacterial growth was observed in samples from the DR‐ZnCuW‐LDH+US (IV) group, consistent with the in vitro antibacterial assay results (Figure 4F). Histological analysis of infected skin tissue was performed using hematoxylin and eosin (H&E) staining on day 10 (Figure 4G). Key histological features were annotated, including intact epidermis (green arrow), inflammatory cell infiltration (red arrow), and new blood vessel formation (yellow arrow). Severe inflammatory cell infiltration and epidermal thickening were observed in the PBS (I) group. The DR‐ZnCuW‐LDH (II) and US (III) groups showed moderate improvement, while the DR‐ZnCuW‐LDH+US (IV) group demonstrated the most intact epidermis, a significant reduction in inflammatory cell infiltration, and enhanced angiogenesis, including the formation of new hair follicles. Gram staining results further confirmed that infection persisted in the PBS (I), DR‐ZnCuW‐LDH (II), and US (III) groups, with extensive Gram‐positive areas observed, particularly in the PBS (I) group (Figure 4H). Masson's trichrome staining revealed that the blue collagen fiber deposition was most prominent in the DR‐ZnCuW‐LDH+US (IV) group (Figure 4I; Figure S20). In addition, the collagen deposition was 6.55% ± 0.86, 6.96% ± 1.11, 11.44 % ± 1.21, 36.84 % ± 1.71 for PBS (I), DR‐ZnCuW‐LDH (II), US (III), and DR‐ZnCuW‐LDH+US (IV) group (Figure S20). These findings highlight the potential of DR‐ZnCuW‐LDH nanosheets as an effective material for sonodynamic antibacterial therapy and wound healing.

Immunohistochemical staining for iNOS, a marker highly expressed during M1 macrophage polarization, facilitates the release of inflammatory mediators [51, 52]. As shown in Figure S21A,B, skin tissue treated with PBS (I) exhibited substantial infiltration of M1‐type macrophages (indicated by extensive brown staining), confirming a severe inflammatory response (9.55% ± 3.51). Treatment with DR‐ZnCuW‐LDH (II) or US (III) resulted in reduced inflammatory expression, indicating a less intense but ongoing inflammatory response (17.86% ± 0.8 and 15.36% ± 1.82, respectively). Following treatment with DR‐ZnCuW‐LDH+US (IV), iNOS levels in skin tissue decreased significantly to 6.50% ± 1.92, indicating that DR‐ZnCuW‐LDH+US effectively mitigates inflammation in infected tissue and facilitates wound healing. Similarly, Arg1, an M2‐type macrophage marker [53] (Figure S21C,D), revealed a small population of densely distributed M2‐type macrophages in the PBS (I) group, indicating a pronounced inflammatory response (4.18% ± 1.48). In contrast, the DR‐ZnCuW‐LDH (II) and US (III) groups exhibited a reduced presence of inflammatory cells, suggesting a less severe but persistent inflammatory state (8.26% ± 1.29 and 7.99% ± 1.27, respectively). Notably, treatment with DR‐ZnCuW‐LDH+US (IV) significantly increased the level of Arg1 in the skin tissue to 33.10% ± 2.36, demonstrating that DR‐ZnCuW‐LDH+US not only alleviates the inflammatory response in infected tissue but also effectively promotes wound healing. These findings collectively underscore the potential of DR‐ZnCuW‐LDH nanosheets as a promising material for sonodynamic antibacterial therapy and accelerated wound healing.

### 2.6 Therapy Evaluation of Bacterial Keratitis In Vivo

**FIGURE 5:**
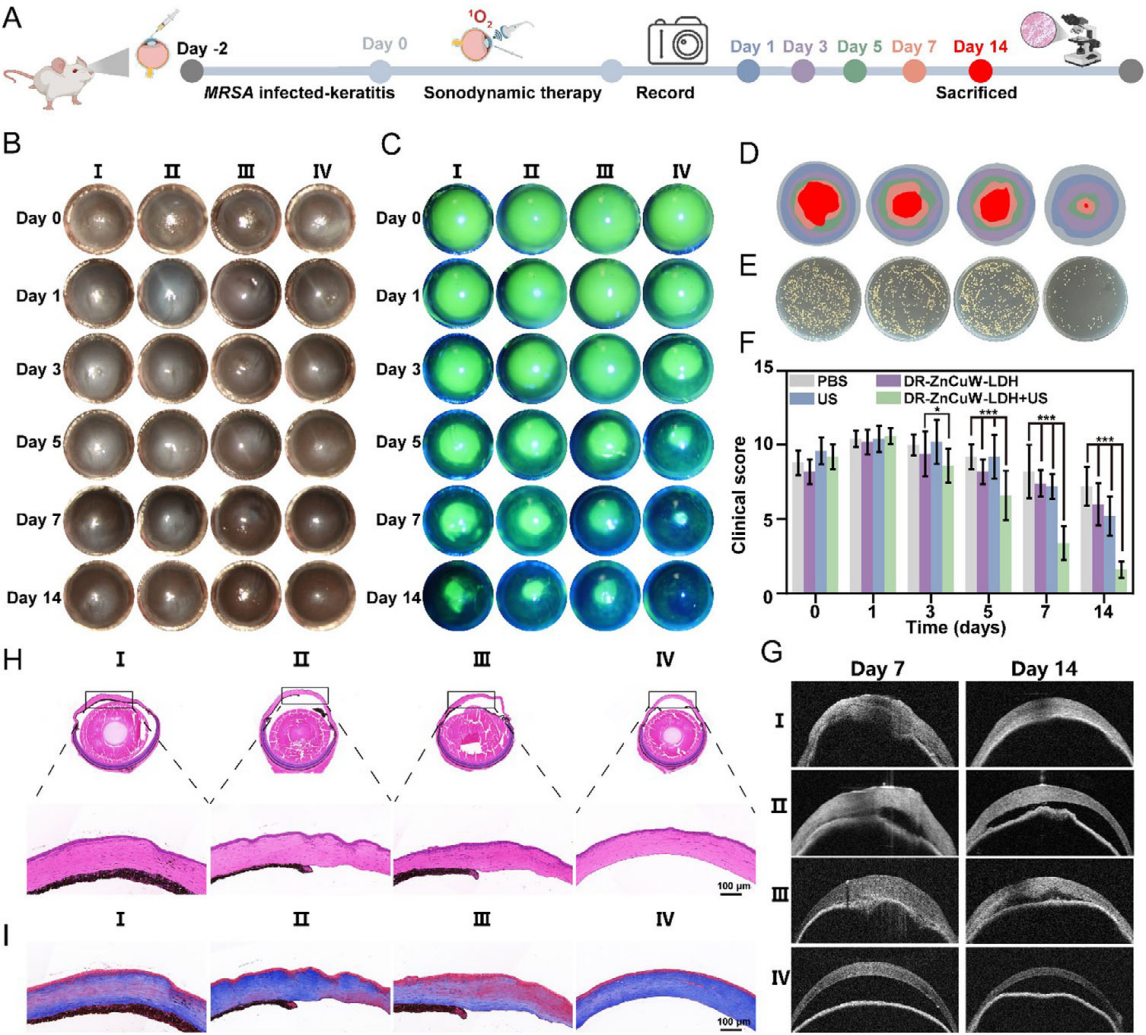
The therapeutic assessment of DR‐ZnCuW‐LDH nanosheets in *MRSA*‐infective keratitis. (A) Schematic illustration of the experimental model timeline from *MRSA* inoculation (day‐2) to final assessment (day 14). (B) Representative photographs of corneal surfaces in four treatment groups (I‐IV: PBS, DR‐ZnCuW‐LDH nanosheets, US, and DR‐ZnCuW‐LDH nanosheets + US, respectively) on days 0, 1, 3, 5, 7, and 14. (C) Corresponding sodium fluorescein staining of corneas at the same time points of (B). (D) The corresponding healing trace of sodium fluorescein staining on keratitis. Gray, blue, purple, green, orange, and red represented abscess sizes on days 0, 1, 3, 5,7, and 14, respectively. (E) Photographs of bacterial colonies from corneal tissues across distinct treatment groups on day 3. (F) Clinical scores of corneal healing in the four groups at the indicated time points (mean ± s.d., n = 6). (G) Representative anterior segment optical coherence tomography (AS‐OCT) images of *MRSA*‐infected keratitis with different treatments on days 7 and 14. (H) H&E and (I) Masson's trichrome staining of corneal sections on day 14. Data are presented as mean ± s.d., ^*^p < 0.05, ^**^p < 0.01, and ^***^p < 0.001.

Antimicrobial dynamic therapy is primarily divided into two categories: exogenous and endogenous. Exogenous therapies mainly include PDT and SDT. Among these, PDT is currently the most extensively studied treatment strategy for bacterial keratitis (BK). However, severe BK often involves bacterial invasion deep into the corneal stroma, where light penetration can be attenuated, and thermal effects may damage sensitive ocular structures. The application of SDT, with its ability to penetrate the corneal layers without thermal injury, offers a distinct advantage. To investigate this, a mouse model of keratitis was developed using MRSA infection, simulating a deep stromal infection environment. To investigate the therapeutic efficacy of DR‐ZnCuW‐LDH nanosheets in BK following SDT treatment, a mouse model of keratitis was developed using *MRSA* infection (Figure 5A). The morphology of the anterior segment of the eye was assessed with a slit‐lamp microscope following different local ocular treatments. Additionally, fluorescein sodium staining was performed to assess corneal epithelial integrity and stromal tissue damage, with corresponding clinical scores recorded. As shown in Figure 5B,C, all mouse corneas exhibited clinical symptoms of *MRSA* infection. On the first day post‐infection, severe purulent ulcers and dense opacities nearly covered the entire cornea, maintaining high clinical total scores, thus confirming the successful establishment of the BK model. Corneal opacity scores were monitored using a slit‐lamp microscope on days 1, 3, 5, 7, and 14 post‐infection. In the PBS (I), DR‐ZnCuW‐LDH (II), and US (III)groups, *MRSA*‐infected corneas exhibited progressive morphological deterioration, including purulent corneal ulcers and severe corneal defects, over the 14‐day observation period. In contrast, treatment with DR‐ZnCuW‐LDH+US (IV) resulted in gradual improvement in corneal symptoms. By day 5, the therapeutic effect of DR‐ZnCuW‐LDH + US (IV) was significantly superior to that of the control groups, with corneal morphology nearly restored to normal by day 14. Correspondingly, the fluorescein staining area and clinical total scores decreased significantly over time (Figure 5D,F).

To further assess antibacterial efficacy, the quantity of viable bacteria in the corneal tissues was measured on day 5. As shown in Figure 5E, the DR‐ZnCuW‐LDH + US group exhibited a significantly reduced bacterial colony count compared to the other three groups, highlighting the enhanced antibacterial effect achieved through the synergistic interaction of US and DR‐ZnCuW‐LDH. Corneal healing was further assessed using anterior segment optical coherence tomography (AS‐OCT) to observe cross‐sectional images of corneal tissues. Changes in corneal thickness were monitored on days 7 and 14 post‐treatment under different conditions. As shown in Figure 5G, severe corneal edema was observed in all groups on day 7, corresponding to the acute inflammatory phase. By day 14, the DR‐ZnCuW‐LDH + US (IV) group exhibited no significant corneal thickening, indicating minimal scarring and the absence of endophthalmitis. This was further supported by the lack of adhesion between the cornea and the anterior chamber angle. In contrast, the PBS (I), DR‐ZnCuW‐LDH (II), and US (III) groups displayed varying degrees of corneal thickening and adhesion between the cornea and the anterior chamber angle.

Histological analyses were performed to further evaluate corneal healing. H&E staining of corneal tissues revealed an intact corneal structure and transparent corneal layers in the DR‐ZnCuW‐LDH + US (IV) group (Figure 5H). Enlarged images showed minimal inflammatory cell infiltration and significantly reduced corneal collagen fiber edema in this group. In contrast, the PBS‐treated group exhibited abnormal corneal structures and extensive inflammatory cell infiltration within the corneal stroma, as observed in H&E staining. Masson's trichrome staining was used to assess collagen activity. Collagen fibers appeared blue or dark blue, while fibrous connective tissues appeared white or light blue. As shown in Figure 5I, during the healing phase of bacterial keratitis, the PBS (I) group displayed extensive fibrous tissue proliferation in the stromal layer, resulting in corneal flap thickening. This disordered collagen distribution could lead to refractive errors that are difficult to correct. In contrast, the DR‐ZnCuW‐LDH + US (IV) group retained most of the collagen, demonstrating superior preservation of corneal architecture. In conclusion, DR‐ZnCuW‐LDH + US (IV) effectively treats *MRSA*‐induced keratitis by enhancing oxidase‐mimicking activity through ultrasound‐activated sonosensitizers. This approach represents a promising novel strategy for the clinical treatment of bacterial keratitis.

### 2.7 Biocompatibility Assessment of DR‐ZnCuW‐LDH In Vivo

**FIGURE 6:**
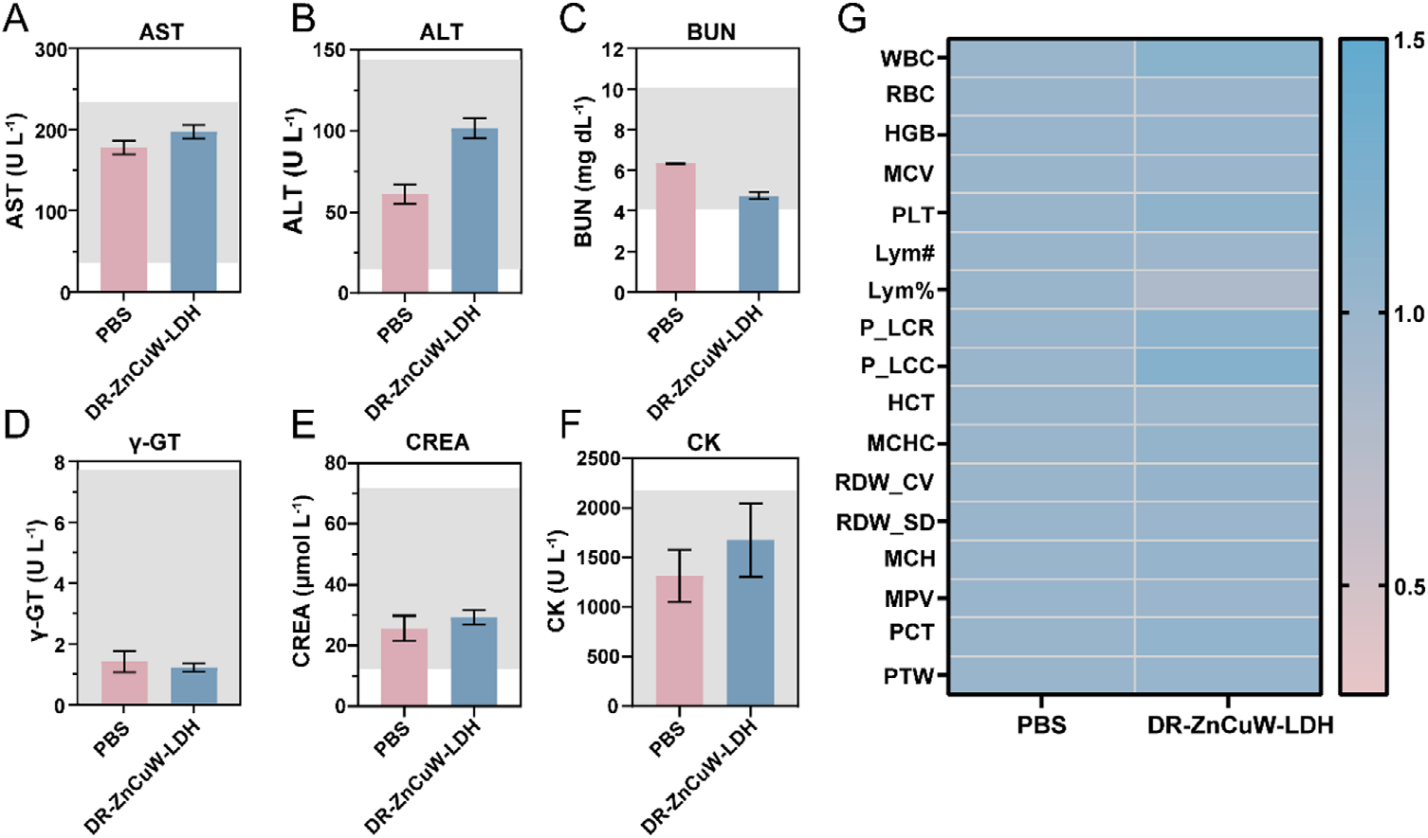
Biosafety evaluations of DR‐ZnCuW‐LDH nanosheets on healthy mice. (A‐F) Blood chemistry of the liver, heart, and kidneys, including aspartate aminotransferase (AST), alanine aminotransferase (ALT), blood urea nitrogen (BUN), γ‐glutamyl transferase (γ‐GT), creatinine (CREA), and creatine kinase (CK). (G) Heatmap of Complete Blood Count (CBC), including white blood cells (WBC), red blood cells (RBC), hemoglobin (HGB), mean corpuscular volume (MCV), platelets (PLT), and other key parameters. Data is represented as mean ± s.d. (n = 3) of three biological replicates, ^*^ p < 0.05, ^**^p < 0.01, and ^***^p < 0.001.

To facilitate the practical application of antibacterial agents, they must exhibit robust efficacy while maintaining minimal toxicity and a strong safety profile. Accordingly, to evaluate the biosafety of DR‐ZnCuW‐LDH nanosheets, we conducted comprehensive in vivo assessments, including major blood biochemical analyses, hemolysis testing, and hematological evaluations. In the hemolysis assay, deionized water served as a positive control (causing hypertonic lysis), and PBS was used as a negative control. Notably, the hemolysis rate at 200 µg mL^−1^was below 5%, indicating no significant hemolytic effect (Figure S22). Given that in vivo biocompatibility is critical for future clinical translation [54, 55], we next examined potential toxicity in a murine model wherein mice were intraperitoneally injected with PBS or DR‐ZnCuW‐LDH for 48 h and then evaluated for physiological changes. As shown in Figure 6A–F, no obvious differences were detected between the PBS and DR‐ZnCuW‐LDH groups in serum levels of aspartate aminotransferase (AST), alanine aminotransferase (ALT), blood urea nitrogen (BUN), γ‐glutamyl transferase (γ‐GT), creatinine (CREA), and creatine kinase (CK), suggesting that DR‐ZnCuW‐LDH nanosheets do not adversely affect blood biochemistry. Similarly, routine hematological markers‐including white blood cells (WBC), red blood cells (RBC), hemoglobin (HGB), platelets (PLT), and lymphocyte count and percentage (Lym#, Lym%)‐remained unchanged following DR‐ZnCuW‐LDH treatment (Figure 6G), confirming negligible hemocompatibility concerns. Furthermore, histopathological examination of major organs revealed no obvious inflammation or tissue damage across various treatment conditions (Figure S23). Taken together, these in vitro and in vivo findings underscore the excellent biocompatibility of DR‐ZnCuW‐LDH nanosheets and highlight their potential as a safe, versatile nanotherapeutic platform.

## 3 Conclusions

Through the ambient‐condition acid‐etching approach enabled defect engineering, we constructed defect‐rich 2D DR‐ZnCuW‐LDH nanosheets featuring abundant OVs, a crystalline‐to‐polycrystalline phase transition, and a narrowed bandgap from 3.29 to 1.80 eV, collectively enhancing e^−^‐h^+^ separation and ultrasound‐triggered ROS production. As a result, DR‐ZnCuW‐LDH delivered approximately fourfold higher ^1^O_2_ output than pristine ZnCuW‐LDH and achieved rapid, near‐complete eradication of *MRSA* and *P. aeruginosa*, alongside robust biofilm disruption under US parameters. In vivo, DR‐ZnCuW‐LDH combined with US accelerated the healing of *MRSA*‐infected wounds and effectively treated *MRSA*‐induced bacterial keratitis with minimal scarring, while exhibiting favorable hemocompatibility and negligible systemic toxicity. Overall, these findings establish DR‐ZnCuW‐LDH as a safe and efficacious inorganic sonosensitizer and highlight defect engineering as a generalizable strategy for next‐generation antimicrobial SDT platforms.

## 4 Experimental Section

### 4.1 Chemicals and Materials

Zinc Nitrate Hexahydrate (Zn (NO_3_)_2_·6H_2_O, 98%), cupric nitrate trihydrate (Cu (NO_3_)_2_·6H_2_O, 99.9%), Sodium Tungstate Dihydrate (Na_2_WO_4_·2H_2_O,> 99.0%), singlet oxygen sensor green (SOSG), 2,2,6,6‐tetramethyl‐4‐piperidone (TEMP), 1,3‐diphenylisobenzofuran, 5,5'‐Dithiobis (2‐nitrobenzoic acid) (DTNB), sodium hydroxide (NaOH, >98.0%) and glutathione (GSH) were purchased from Aladdin Reagent (Shanghai, China). o‐nitrophenyl β‐D‐galactopyranoside (ONPG) was obtained by Beyotime Biotechnology Co., Ltd. (Shanghai, China). 3,3’‐dipropylthiadicarbocyanine Iodide (DiSC3(5)) was provided by Maokang Biotechnology Co., Ltd (Shanghai, China). SYTO‐9/PI Live/Dead Bacterial Viability Kits were obtained from Invitrogen (USA). 2’,7’‐Dichlorofluorescin Diacetate (DCFH‐DA), Agar powder, tryptone soya broth (TSB), and LB agar were purchased from Solarbio (Beijing, China). All chemical reagents were purchased from commercial companies and used as received without additional purification.

### 4.2 Characterizations

Transmission electron microscopy (TEM) images were captured from a JEM 2100 Plus Microscope (EI Talos F200S G, FEI) operating at 200 kV. X‐ray diffraction (XRD) patterns were performed by a scan rate of 2 °/min over the 2θ range of 10–60° (D8 ADVANCE, Bruker). X‐ray photoelectron spectroscopy (XPS) (ESCALAB, 250Xi, Thermo Fisher Scientific, USA) was used to evaluate the state of element valence states of ZnCuW‐LDH and DR‐ZnCuW‐LDH nanosheets. The thickness of nanosheets was characterized by an Atomic Force Microscope (AFM) (Dimension Icon, Bruker). Reactive oxygen species (ROS) were measured utilizing electron spin resonance (ESR) investigations using a Bruker EMX A300 spectrometer. Hydrodynamic sizes and zeta potentials were determined using a Zetasizer UV spectrometer (Malvern Instruments, UK).

### 4.3 Synthesis of ZnCuW‐LDH Nanosheets

2 mmol (0.5950 g) of Zn (NO_3_)2·6H_2_O, 0.05 mmol (0.01208 g) of Na_2_WO_4_·2H_2_O, and 0.95 mmol (0.3134 g) of Cu (NO_3_)2·6H_2_O were dissolved in 30 mL of deoxygenated deionized water to prepare solution A. 2 g of NaOH was weighed and dissolved in 50 mL of deoxygenated deionized water to prepare solution B. Under a nitrogen atmosphere in an oil bath at 80°C, solution B was slowly added dropwise into solution A until the pH reached 9.7. The mixture was stirred for 20 min and then transferred to a reaction vessel for hydrothermal treatment at 80°C for 24 h. The resulting product was centrifuged and washed with DI water to obtain ZnCuW‐LDH nanosheets.

### 4.4 Preparations of DR‐ZnCuW‐LDH Nanosheets

The synthesized ZnCuW‐LDH was incubated in a buffer solution at pH 4.0 for 4 h. The resulting DR‐ ZnCuW ‐LDH was subsequently separated by centrifugation (8000 × g, 5 min) and re‐dispersed in chilled DI water.

### 4.5 ROS Detection of ZnCuW‐LDH and DR‐ZnCuW‐LDH Nanosheets

The ESR spectrometer was used to determine the ROS generation of ZnCuW‐LDH and DR‐ZnCuW‐LDH nanosheets by using TEMP as a trapper to capture ^1^O_2_. In summary, 100 µL of TEMP (90 mM) was mixed with 100 µL of ZnCuW ‐LDH or DR‐ZnCuW‐LDH solution (50 µg mL^−1^), and the generation of ^1^O_2_ was analyzed by ESR under ultrasound for 5 min. The ESR measurement parameters were as follows: a scan time of 41.9 s, a scan width of 200 G, a microwave power of 2.015 mW, and a microwave frequency of 9.873 GHz.

### 4.6 GSH‐Depleted Property of DR‐ZnCuW‐LDH Nanosheets

The depletion of GSH was monitored using a DTNB probe by measuring its absorbance at 412 nm. Specifically, a GSH solution (0.1 M) prepared in PBS was combined with varying concentrations of 100 µL DR‐ZnCuW‐LDH solution (50 µg mL^−1^) at room temperature. At predetermined time intervals, 100 µL of the reaction mixture was diluted into 900 µL of phosphate‐buffered saline (PBS, pH 7.4). Subsequently, DTNB (0.1 mM) was added to the solution, and the mixture was incubated for 3 min. The absorbance of the resulting solution was then recorded at 412 nm. The extent of GSH oxidation was calculated using the corresponding formula.

$$\mathrm{Loss}\;\mathrm{of}\;\mathrm{GSH}\;\left(\%\right)=\frac{OD_{\mathrm{control}}\;-\;OD_{\mathrm{sample}}\;}{OD_{\mathrm{control}}}\times 100\%$$

### 4.7 Cell Culture and Bacterial Culture

L929 and HCEC cell lines were cultured in a humidified incubator at 37°C with 5% CO_2_. The high‐glucose Dulbecco's modified Eagle medium (DMEM; Gibco, USA), supplemented with 10% fetal bovine serum (FBS) and 1% penicillin‐streptomycin, was used to maintain the cells. *MRSA* or POA1 were grown in a suitable nutrient medium (TSB or LB) at 37°C under aerobic conditions. For liquid cultures, bacterial suspensions were incubated with shaking at 200 rpm to ensure proper aeration. The bacterial density was monitored by measuring the optical density at 600 nm (OD 600) using a spectrophotometer. For solid cultures, bacteria were streaked on agar plates and incubated overnight.

### 4.8 Stability of DR‐ZnCuW‐LDH Nanosheets

The dispersions of DR‐ZnCuW‐LDH nanosheets were prepared in three different media: PBS, deionized water, and DMEM medium (supplemented with 10% FBS). The DR‐ZnCuW‐LDH nanosheets were dispersed in each of the three media at a concentration of 50 µg mL^−1^. The particle size distribution of the DR‐ZnCuW‐LDH nanosheets was assessed using dynamic light scattering (DLS) for 2 weeks.

### 4.9 Intracellular ROS Detection in Bacteria

The intracellular ROS production induced by DR‐ZnCuW‐LDH was assessed using the DCFH‐DA probe. Briefly, 1 mL aliquots of *MRSA* and *P. aeruginos*a suspensions (∼1×10^8^ CFU/mL in PBS) were incubated at 37°C for 4 h with one of the following: (1) PBS +bacteria, (2) PBS +bacteria + US (5 min), (3) PBS +bacteria + US (10 min), (4) DR‐ZnCuW‐LDH +bacteria, (5) DR‐ZnCuW‐LDH +bacteria + US (5 min), (6) DR‐ZnCuW‐LDH +bacteria + US (10 min). The DR‐ZnCuW‐LDH solution was prepared at a concentration of 50 µg mL^−1^. Following treatment, the cells were incubated with DCFH‐DA for 30 min at 37°C, then pelleted and washed twice to remove excess probe. The bacterial pellets were subsequently concentrated and resuspended in 10 µL PBS. Confocal laser scanning microscopy (CLSM) was used to capture fluorescence images, and fluorescence intensities were quantified using ImageJ.

### 4.10 Evaluation of Membrane Potential

The disruption of bacterial membrane depolarization was monitored using the potential‐sensitive dye DiSC_3_(5). Briefly, *MRSA* and *P. aeruginosa* suspensions (∼1×10^8^ CFU/mL) were washed twice and diluted tenfold in 5 mM HEPES containing 20 mM glucose. The diluted cells were equilibrated with 0.4 µM DiSC_3_(5) at 37°C for 1 h to allow dye partitioning into the membrane. Subsequently, (1) PBS + bacteria; (2) PBS + bacteria + US (5 min); (3) PBS + bacteria + US (10 min); (4) DR‐ZnCuW‐LDH + bacteria; (5) DR‐ZnCuW‐LDH + bacteria + US (5 min); (6) DR‐ZnCuW‐LDH + bacteria + US (10 min) were added. A DR‐ZnCuW‐LDH solution was prepared at a concentration of 50 µg mL^−1^. After 5 min, fluorescence intensity (FI) was recorded on a microplate reader (excitation 622 nm, emission 670 nm). HEPES buffer and 2.5% Triton X‐100 served as the negative and positive controls, respectively. Relative fluorescence intensity (FI) was calculated as: Relative FI (%) =(FIsample‐FI_HEPES_)/(FI_Triton X‐100_‐FI_HEPES_) 100%.

### 4.11 ONPG Hydrolysis

Membrane permeability was quantified via the ONPG hydrolysis assay. In brief, bacterial suspensions *of MRSA* and *P. aeruginosa* (∼1×10^^8^ CFU/mL) were exposed to (1) PBS +bacteria; (2) PBS +bacteria + US (5 min); (3) PBS +bacteria + US (10 min); (4) DR‐ZnCuW‐LDH +bacteria; (5) DR‐ZnCuW‐LDH +bacteria + US (5 min); (6) DR‐ZnCuW‐LDH +bacteria + US (10 min) at 37°C for 4 h. The DR‐ZnCuW‐LDH solution was prepared at a concentration of 50 µg mL^−1^. Thereafter, 15 µL of the treated suspension was mixed with 15 µL ONPG (12.5 mM) and 120 µL deionized water and incubated for 30 min at 37°C. Absorbance at 420 nm was then measured on a microplate reader.

### 4.12 Protein Leakage Assay

To corroborate bacterial membrane disruption, extracellular protein released from *MRSA* and *P. aeruginosa* after the respective treatments was quantified using a bicinchoninic acid (BCA) colorimetric assay. Absorbance at 562 nm was measured with a microplate reader (OD = 562 nm), and the extent of protein leakage was calculated from the corresponding standard calibration curve.

### 4.13 Hemolysis Assay

The hemocompatibility of the material was evaluated by incubating the DR‐ZnCuW‐LDH solution with red blood cells (RBCs) in physiological saline. After incubation for 3 h, the supernatant was collected (3000 rpm, 15 min), and hemoglobin release was measured at 540 nm to calculate the hemolysis percentage. A hemolysis rate below 5% is considered biocompatible.

### 4.14 Antimicrobial Effect of DR‐ZnCuW‐LDH Nanosheets In Vitro

*MRSA*(Mu50) was selected as the Gram (+) bacterial cell strain, while *Pseudomonas aeruginosa* (*P. aeruginosa*) served as the model Gram (‐) bacterial. Once the bacterial density reached an OD of 0.5 at 600 nm, the bacterial suspension was divided into six groups: (1) PBS +bacteria; (2) PBS +bacteria + US (5 mins); (3) PBS +bacteria + US (10 mins); (4) DR‐ZnCuW‐LDH +bacteria; (5) DR‐ZnCuW‐LDH +bacteria + US (5 mins); (6) DR‐ZnCuW‐LDH +bacteria + US (10 mins). All groups were prepared in sterilized PBS. The antibacterial activity of DR‐ZnCuW‐LDH nanosheets was assessed using the spread plate method. Briefly, a bacterial suspension with a concentration of 1×10^8^ CFU mL^−1^ and DR‐ZnCuW‐LDH nanosheets (50 µg mL^−1^) was mixed in 1 mL PBS. Groups (2), (3), (5), and (6) were additionally exposed to ultrasound (1.0 W cm^−2^) for 5 or 10 min, and all groups were incubated at 37°C for 2 h. After incubation, 20 µL of the diluted bacterial suspension was spread onto agar plates and incubated at 37°C for 16 h. Finally, the number of bacterial colonies was determined using the plate counting method.

### 4.15 Bacterial Live/Dead Stain

Bacterial cultures are treated with SYTO‐9 and PI for roughly 20 min. Following incubation, the cultures are washed three times with phosphate‐buffered saline (PBS) to remove excess dye. The stained samples are analyzed using a confocal laser‐scanning microscope, which enables the differentiation of cell viability based on fluorescence. Live cells emit green fluorescence from SYTO‐9, while dead cells emit red fluorescence due to PI.

### 4.16 Bacterial Biofilm Disruption Assay

To prepare planktonic bacteria, 20 µL of a 1.0×10^8^ CFU mL^−1^ *MRSA* suspension was added to 1.0 mL of TSB medium in a 24‐well plate and cultured overnight at 37°C to form an intact biofilm. Similarly, all groups were designed to evaluate biofilm disruption: (1) PBS +bacteria; (2) PBS +bacteria + US (5 mins); (3) PBS +bacteria + US (10 mins); (4) DR‐ZnCuW‐LDH +bacteria; (5) DR‐ZnCuW‐LDH +bacteria + US (5 mins); (6) DR‐ZnCuW‐LDH +bacteria + US (10 mins). The DR‐ZnCuW‐LDH solution was prepared at a concentration of 50 µg mL^−1^. After treatment, the supernatant from each well was removed, and the remaining *MRSA* biofilm was fixed by drying. The biofilm was then stained with 0.1% crystal violet dye for 15 min, rinsed twice with PBS, and air‐dried at room temperature for 2 h. Finally, the crystal violet‐stained biofilms were dissolved in glacial acetic acid, and the absorbance was measured at 540 nm. Additionally, the integrity of the *MRSA* biofilm was confirmed using the SYTO‐9/PI live/dead staining kit.

### 4.17 Animal Experiments

Animal protocol was carried out in strict accordance with the requirements approved by the Animal Care and Ethics Committee of Wenzhou Medical University. All animal handling procedures adhered to the guidelines established by the Society for the Study of Vision and Ophthalmology.

### 4.18 Subcutaneous Abscess Bacterial Infection Model

Male Balb/c mice (6–8 weeks, 20 g) were subcutaneously infected with *MRSA* on the rear back. After 24 h, the mice were randomly assigned to four groups (n = 3 per group): (1) PBS; (2) DR‐ZnCuW‐LDH; (3) US; (4) DR‐ZnCuW‐LDH + US. For groups (2) and (4), DR‐ZnCuW‐LDH (100 µL, 50 µg mL^−1^) was injected directly into the abscess site. Groups (3) and (4) were treated with US (1.0 W cm^−2^). After 10 days of treatment, all mice were sacrificed, and the wound areas were collected for analysis. Main organs were also excised for further examination. The infected skin tissue was analyzed using H&E staining, immunofluorescence assays, and Masson staining. Additionally, major organs, including the liver, spleen, kidneys, heart, and lungs, were collected and evaluated using H&E staining.

### 4.19 Establishment of Bacterial Keratitis Model

To establish a bacterial keratitis model, healthy C57 mice (6–8 weeks, 20 g) were selected. The corneal epithelium was carefully scratched using a sterile blade or needle to create a superficial injury, ensuring minimal damage to the underlying stroma. An *MRSA* suspension was then applied to the injured corneal surface. The eye was gently massaged to ensure the even distribution of the bacteria. After 24 h, the mice were randomly assigned to four groups (n = 6 per group): (1) PBS; (2) DR‐ZnCuW‐LDH; (3) US; (4) DR‐ZnCuW‐LDH + US. For groups (2) and (4), DR‐ZnCuW‐LDH (50 µL, 50 µg mL^−1^) was injected directly into the abscess site. To assess treatment efficacy, slit‐lamp biomicroscope and sodium fluorescein staining (0.5 mL, 5%) were performed on days 0, 3, 5, 7, and 10 post‐infections. The degree of corneal repair was evaluated by scoring clinical features of *MRSA* keratitis based on three criteria: corneal opacity area, opacity density, and surface regularity. Each feature was graded on a scale of 0 to 4, with a normal, untreated cornea scoring 0 in all categories. The total score for each eye treated ranged from 0 to 12, reflecting the severity of keratitis and the effectiveness of the treatment.

### 4.20 Statistical Analysis

All experiments were repeated at least three times. Data were presented as the mean ± standard deviation and analyzed using GraphPad Prism software (version 9.0) and Origin 2021. Statistical significance was determined using two‐tailed Student's t‐tests and one‐way ANOVA, with significance thresholds set at ^*^ *p < 0.05*, ^**^ *p < 0.01*, and ^***^ *p < 0.001*.

## Funding

Science Fund for Distinguished Young Scholars of Zhejiang Province LR23C100001.

## Conflicts of Interest

The authors declare no conflicts of interest.

## Supporting information




**Supporting File**: advs73919‐sup‐0001‐SuppMat.docx.

## Data Availability

The data that support the findings of this study are available from the corresponding author upon reasonable request.
