# Supplementary material for: Defect‐Rich 2D Layered Double Hydroxides Enhance Sonodynamic Antibacterial Therapy
Source: Adv Sci (Weinh). 2026 Jan 20;13(18):e24216. doi: 10.1002/advs.202524216 (PMC13042360; doi:10.1002/advs.202524216)
Supplement: Supplementary file 1 — Supporting File: advs73919‐sup‐0001‐SuppMat.docx. [file ADVS-13-e24216-s001.docx]

Supporting Information

**Defect-Rich 2D Layered Double Hydroxides Enhance Sonodynamic Antibacterial Therapy**

Qian Liu^1,2#^, Yu Yang^3#^, Rui Zhao^1,2^, Linwei Huang^1,2^, Xingyu Qi^3^, Min Wu^2*^, Jianliang Shen^1,2*^

^1^ Zhejiang Key Laboratory of Ophthalmic Drug Discovery and Medical Device Research, Eye Hospital, Wenzhou Medical University, Wenzhou 325027, China

^2^ Zhejiang Engineering Research Center for Tissue Repair Materials, Wenzhou Institute, University of Chinese Academy of Sciences, Wenzhou, Zhejiang 325001, P. R. China

^3^ State Key Laboratory of Chemical Resource Engineering, Beijing Advanced Innovation Center for Soft Matter Science and Engineering, Beijing University of Chemical Technology, Beijing, 100029 P. R. China

AUTHOR INFORMATION

All authors have approved the final version of this manuscript.

#These authors contributed equally to this work.

*To whom correspondence should be addressed: Jianliang Shen (sjl1@wmu.edu.cn, shenjl@wiucas.ac.cn) and Min Wu (miw100@ucas.ac.cn).


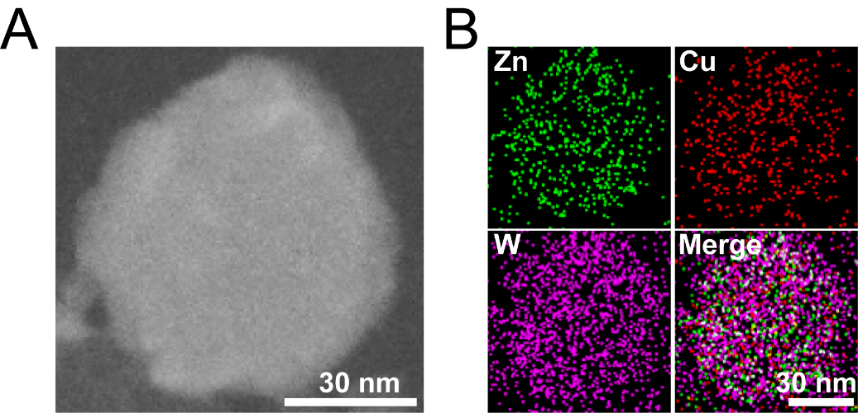


**Figure S1.** EDX characterization of ZnCuW-LDH nanosheets.


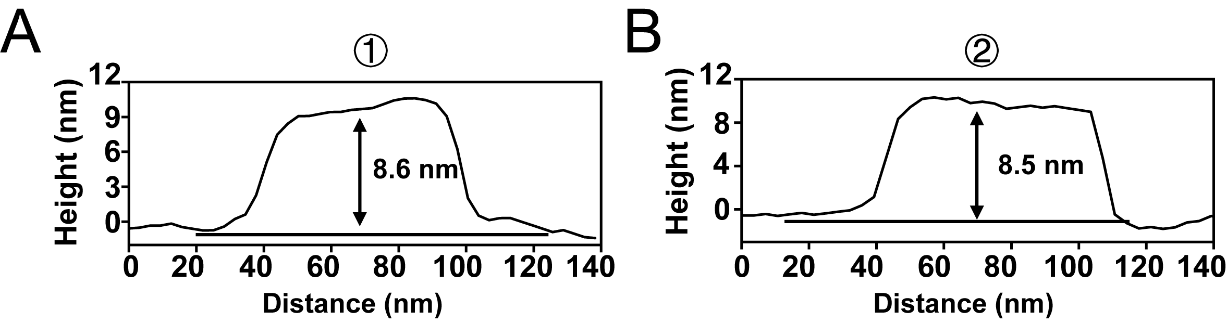


**Figure S2.** AFM characterization of ZnCuW-LDH nanosheets. AFM height profiles correspond to AFM height images of ZnCuW-LDH nanosheets in Figure 1B.


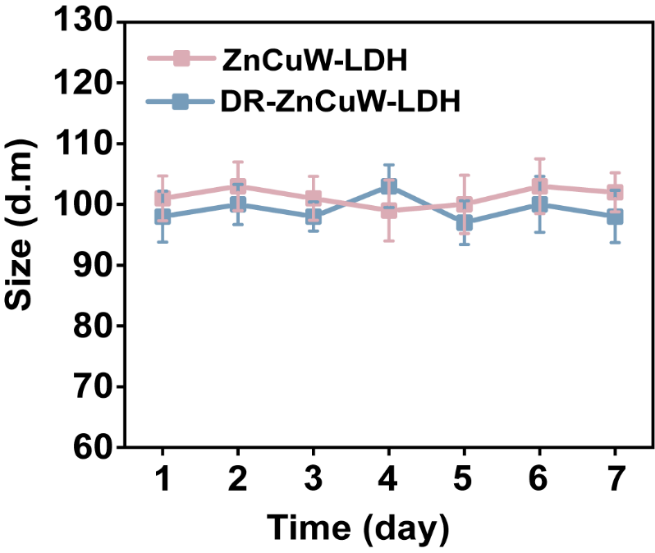


**Figure S3.** DLS characterization of ZnCuW-LDH and DR-ZnCuW-LDH nanosheets. Stability measurements of ZnCuW-LDH and DR-ZnCuW-LDH nanosheets in water by continuously tested for 7 days. Data are presented as mean ± s.d. (n = 3).


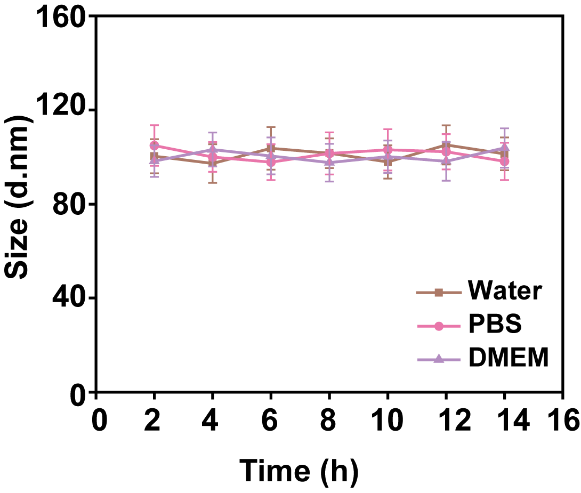


**Figure S4.** DLS characterization of DR-ZnCuW-LDH nanosheets. Stability measurements of DR-ZnCuW-LDH nanosheets in water, PBS, and DMEM were continuously tested for 2 weeks. Data are presented as mean ± s.d. (n = 3).


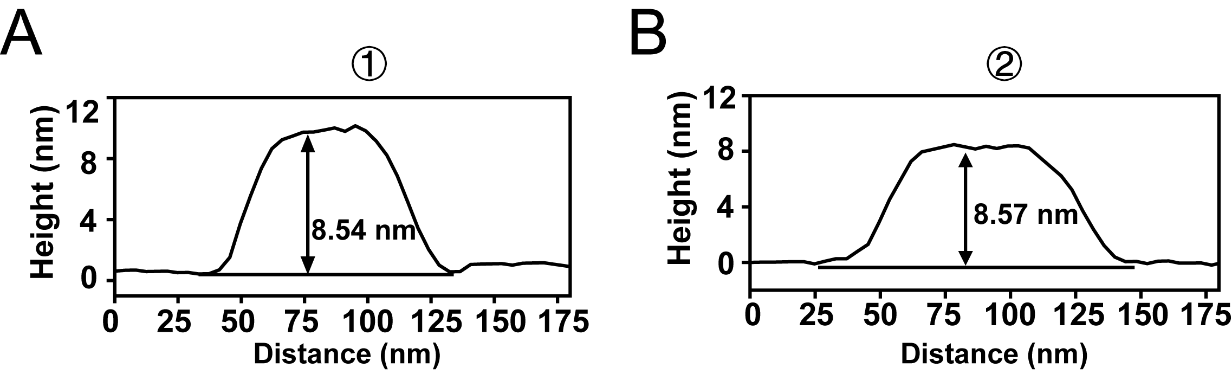


**Figure S5.** AFM characterization of DR-ZnCuW-LDH nanosheets. Corresponding AFM height profiles of DR-ZnCuW-LDH nanosheets in Figure 1E.

**
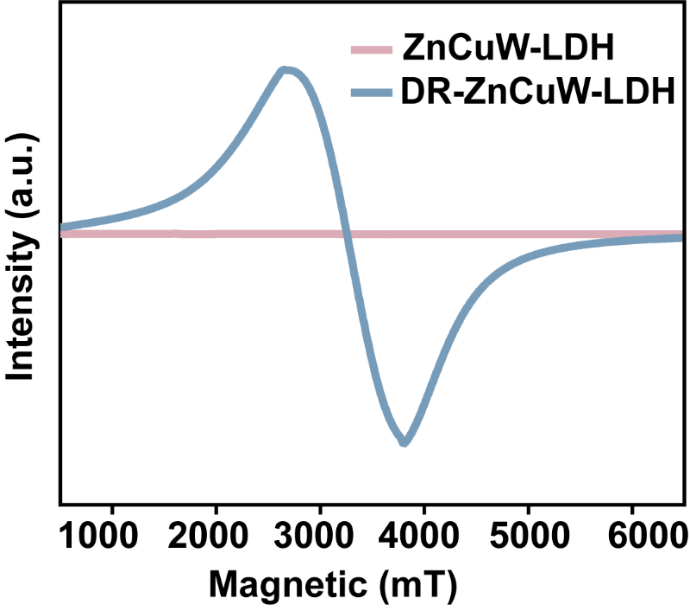
**

**Figure S6.** ESR spectra of ZnCuW-LDH and DR-ZnCuW-LDH nanosheets at *G* = 2.2.


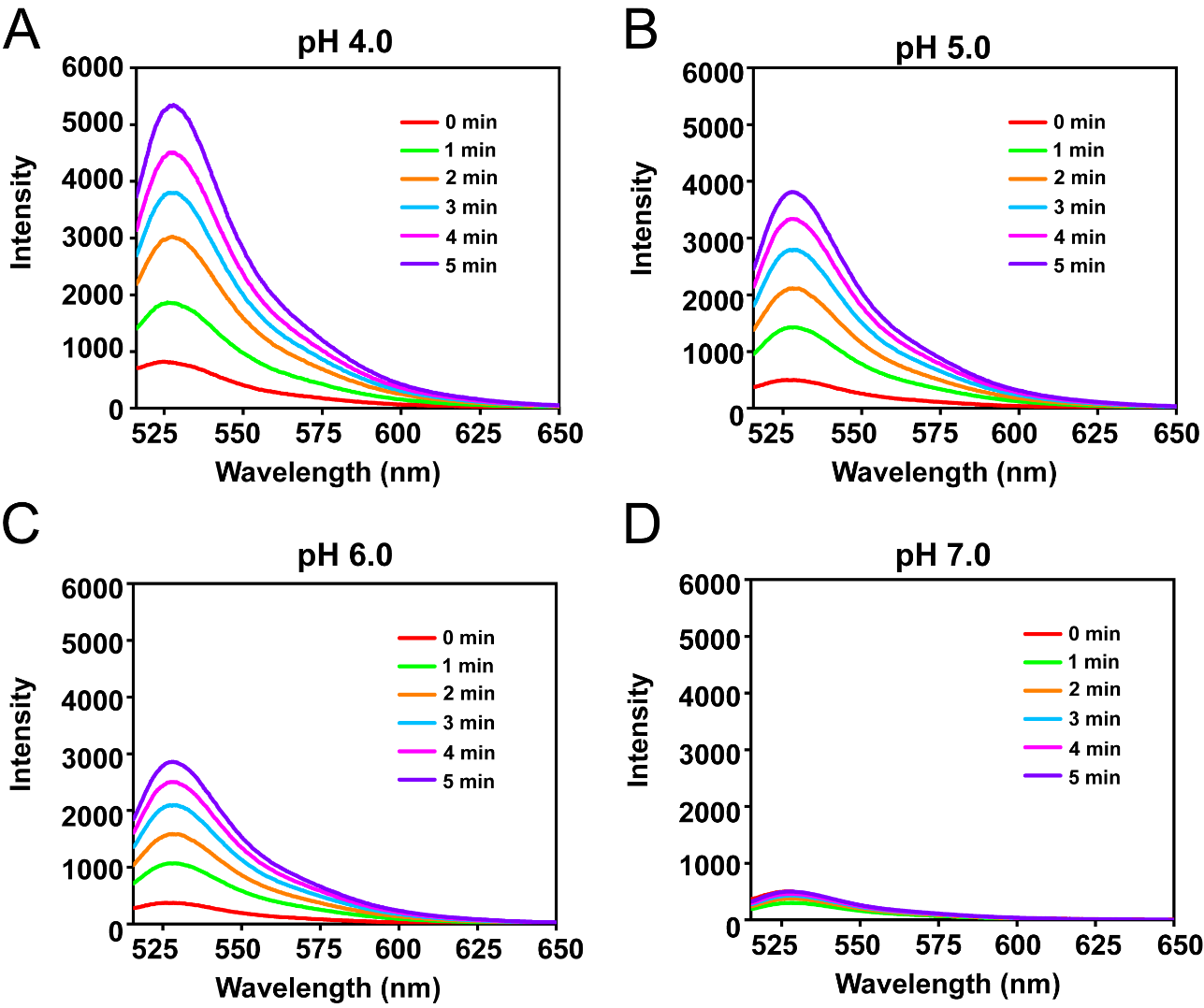


**Figure S7.** Detection of ROS via SOSG assay at different pH conditions. (A) pH=4.0. (B) pH=5.0 (C) pH=6.0. (D) pH=7.0.


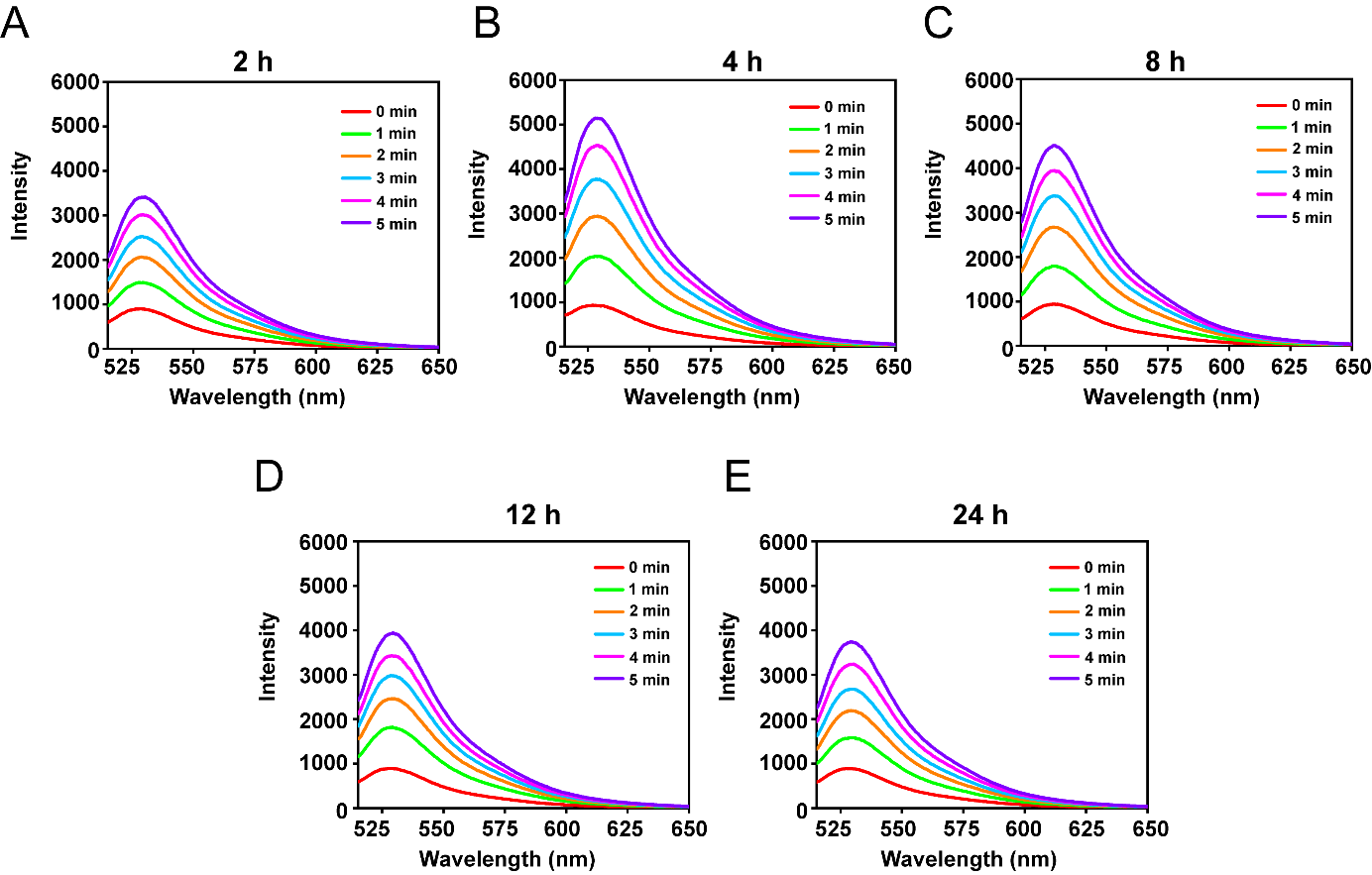


**Figure S8.** Detection of ROS via SOSG assay. The fluorescence spectra of SOSG were recorded in the presence of DR-ZnCuW-LDH nanosheets etched at pH 4.0 for varying durations (2h, 4h, 8h, 12h, and 24h).

**
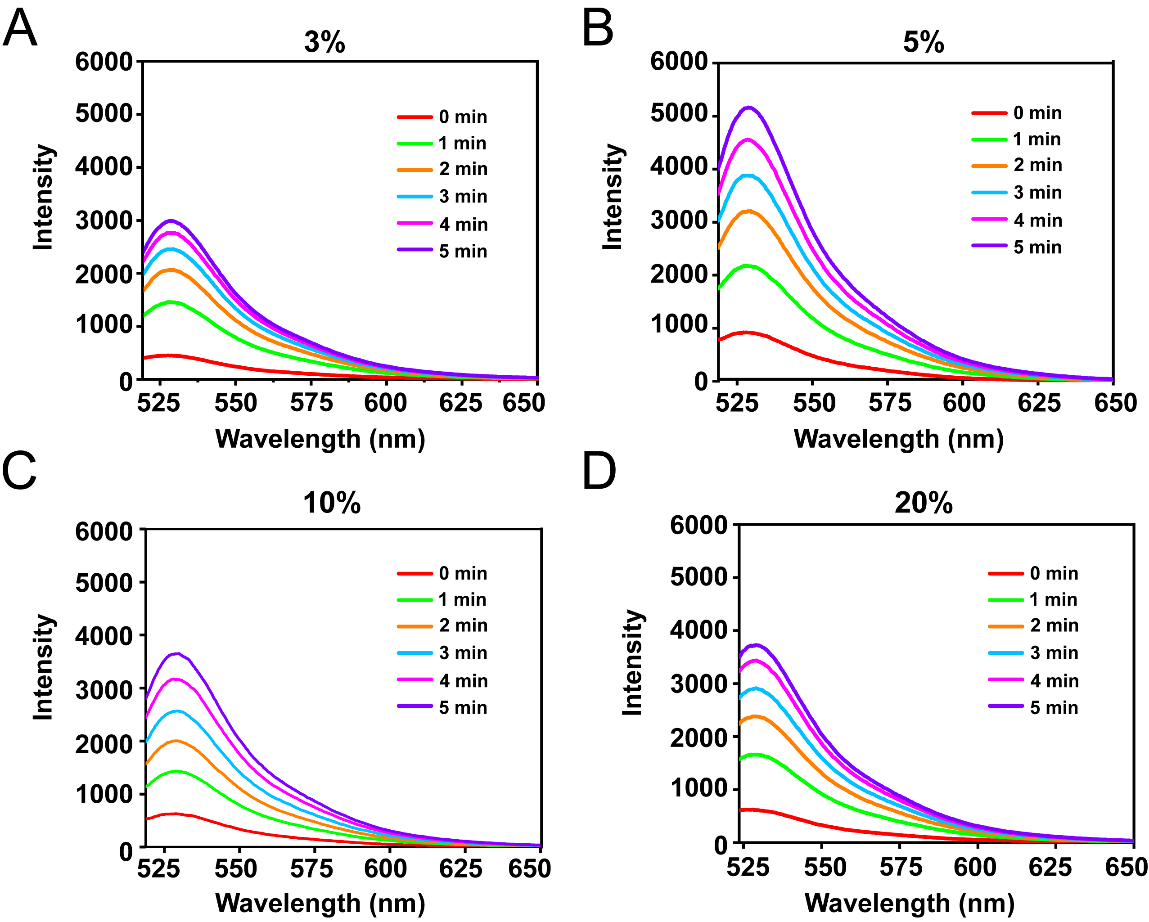
**

**Figure S9.** Detection of ROS via SOSG assay. Effect of Cu doping ratios on ultrasound-induced ROS production via SOSG assay.  (A-D) 3%, 5%, 10% and 20% Cu-dopped.


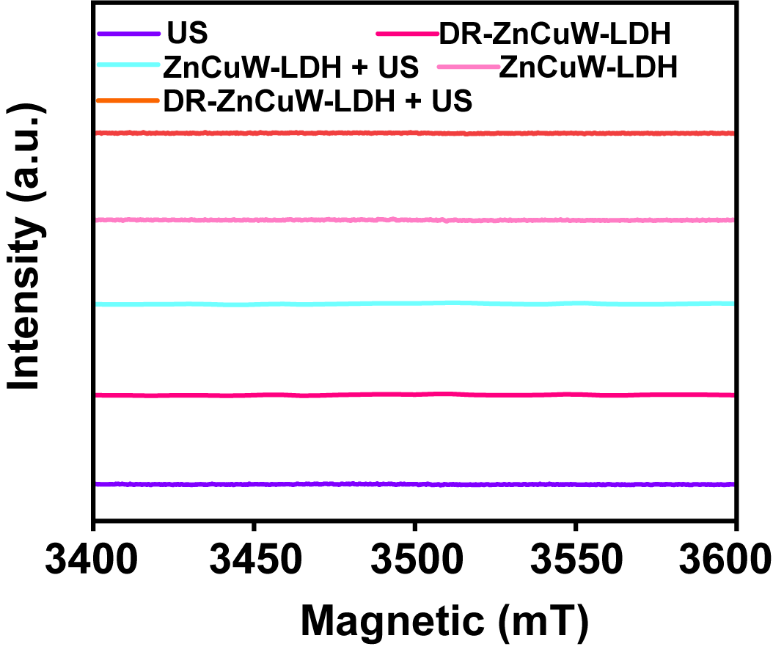


**Figure S10.** Detection of ROS via ESR spectrometer. ESR spectra of ·OH with ZnCuW-LDH and DR- ZnCuW-LDH nanosheets under US irradiation.


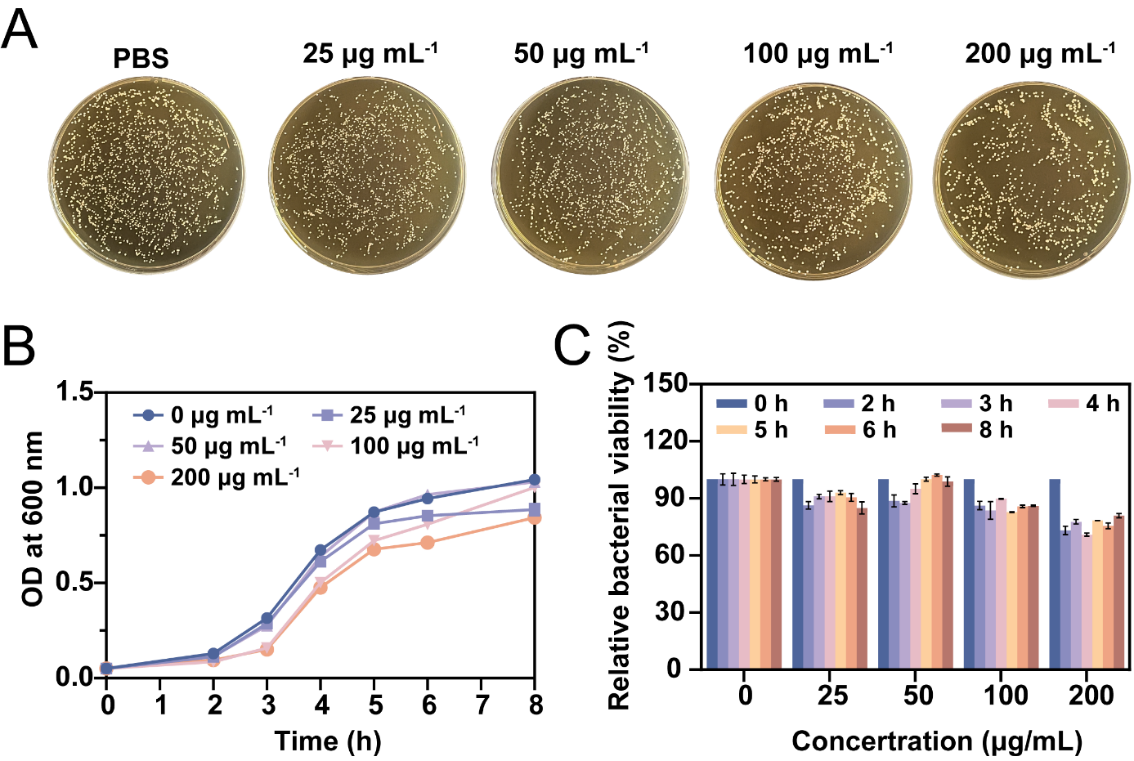


**Figure S11.** In vitro antibacterial effect of individual DR-ZnCuW-LDH nanosheets towards *MRSA*. (A) Argar plates of DR-ZnCuW-LDH nanosheets incubated with *MRSA* for 2h. (B) Bacterial growth curves under various conditions. (C) Relative bacterial viability of *MRSA* after incubation for different time points. Data are presented as mean ± s.d. (n = 3).


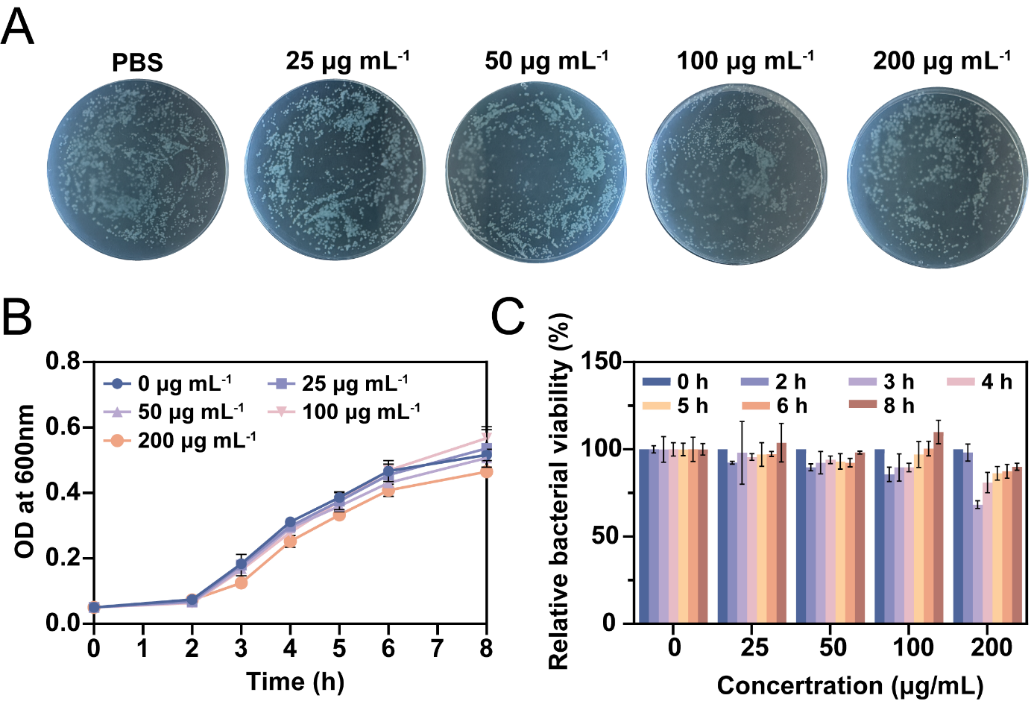


**Figure S12.** In *vitro* antibacterial effect of isolated DR-ZnCuW-LDH nanosheets against *P. aeruginosa*. (A) Argar plates of DR-ZnCuW-LDH nanosheets incubated with *P. aeruginosa* for 2h. (B) Bacterial growth curves under various conditions. (C) Relative bacterial viability of *P. aeruginosa* after incubated for different time points. Data are presented as mean ± s.d. (n = 3).


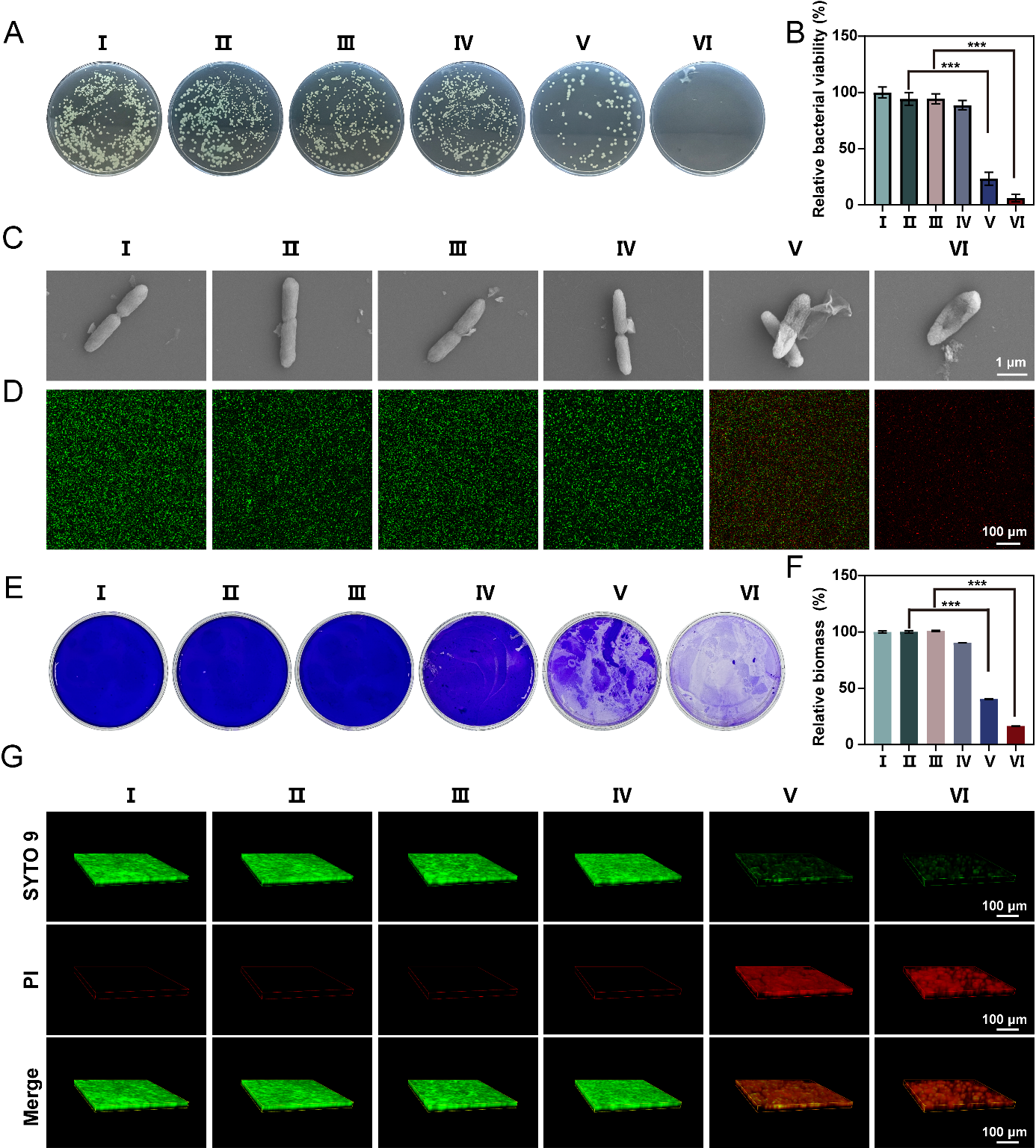


**Figure S13.** Antibacterial and anti-biofilm activities of DR-ZnCuW-LDH nanosheets in *vitro*. (A) Representative agar plates and (B) relative bacterial viability quantified from colony counts after indicated treatments. PBS (I), PBS +US 5min (Ⅱ), PBS +US 10min (Ⅲ), DR-ZnCuW-LDH nanosheets (Ⅳ), DR-ZnCuW-LDH nanosheets + US 5min (V), and DR-ZnCuW-LDH nanosheets + US 10min (VI). (C) SEM images of *P. aeruginosa* morphology under different treatments. (D) Live/dead fluorescence staining of *P. aeruginosa* by given treatments. (E) Crystal violet staining of *P. aeruginosa* biofilms formed under each treatment, and (F) corresponding quantitative analysis of biofilm biomass. (G) CLSM images of SYTO 9 (green)/PI (red)-stained biofilms. Data are presented as mean ± s.d. (n = 3), **p* < 0.05, ***p* < 0.01, and ****p* < 0.001.

**
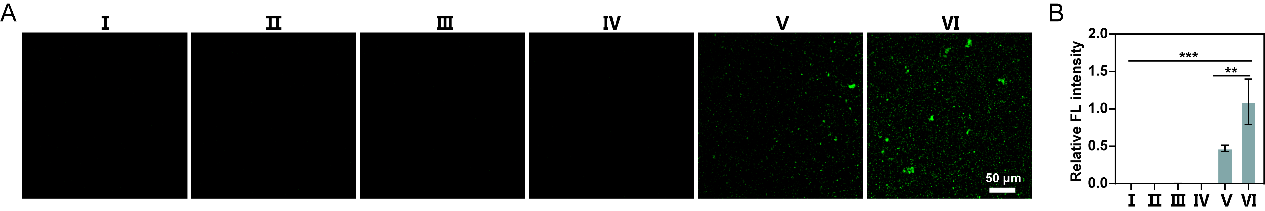
Figure S14.** Bacterial intracellular ROS levels under DR-ZnCuW-LDH with US irradiation. (A) CLSM images of *MRSA* after treatment with DR-ZnCuW-LDH with/without US. PBS (I), PBS +US 5min (Ⅱ), PBS +US 10min (Ⅲ), DR-ZnCuW-LDH nanosheets (Ⅳ), DR-ZnCuW-LDH nanosheets + US 5min (V), and DR-ZnCuW-LDH nanosheets + US 10min (VI). (B) Quantification of intracellular ROS by measuring DCFH-DA relative fluorescence intensity (mean ± SD) for *MRSA* across independent replicates. Data are presented as mean ± s.d. (n = 3), **p* < 0.05, ***p* < 0.01, and ****p* < 0.001.

*
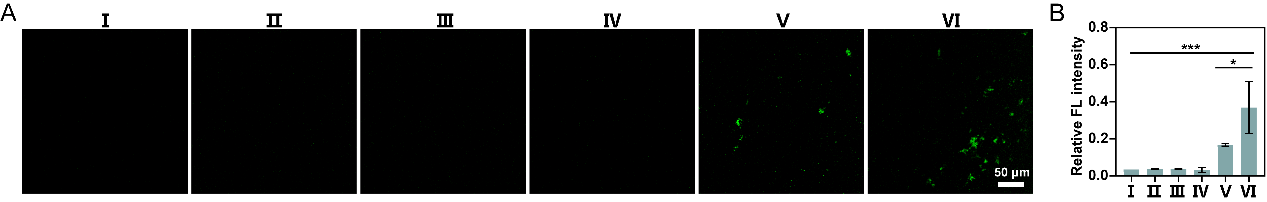
***Figure S15.** The intracellular ROS levels in *P. aeruginosa* upon DR‑ZnCuW‑LDH with US irradiation. (A) CLSM images of P. aeruginosa after various treatments. (B) Quantification of intracellular ROS by measuring DCFH-DA relative fluorescence intensity (mean ± SD) for P. aeruginosa across independent replicates. All data are representative of three independent experiments, **p* < 0.05, ***p* < 0.01, and ****p* < 0.001.

**
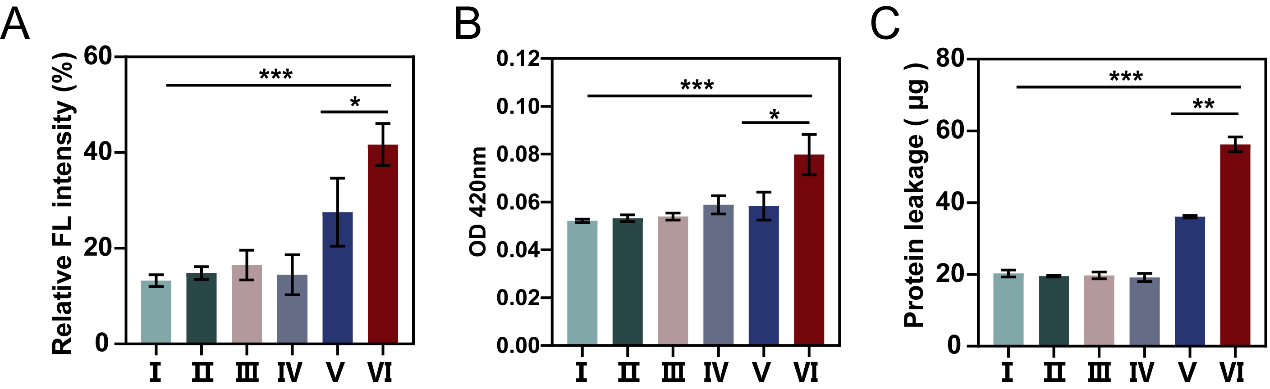
**

**Figure S16.** Potential mechanism of the antibacterial activity of DR-ZnCuW-LDH nanosheets with US irradiation. (A) Membrane potential changes of *MRSA* after different treatments detected by DiSC_3_(5) dye. (B) Membrane permeability of *MRSA* in various groups evaluated with the ONPG hydrolysis assay. (C) The protein leakage from *MRSA* after different treatments using the BCA protein assay kit. PBS (I), PBS +US 5min (Ⅱ), PBS +US 10min (Ⅲ), DR-ZnCuW-LDH nanosheets (Ⅳ), DR-ZnCuW-LDH nanosheets + US 5min (V), and DR-ZnCuW-LDH nanosheets + US 10min (VI). Data are presented as mean ± s.d. (n=3), **p* < 0.05, ***p* < 0.01, and ****p* < 0.001.

.

***
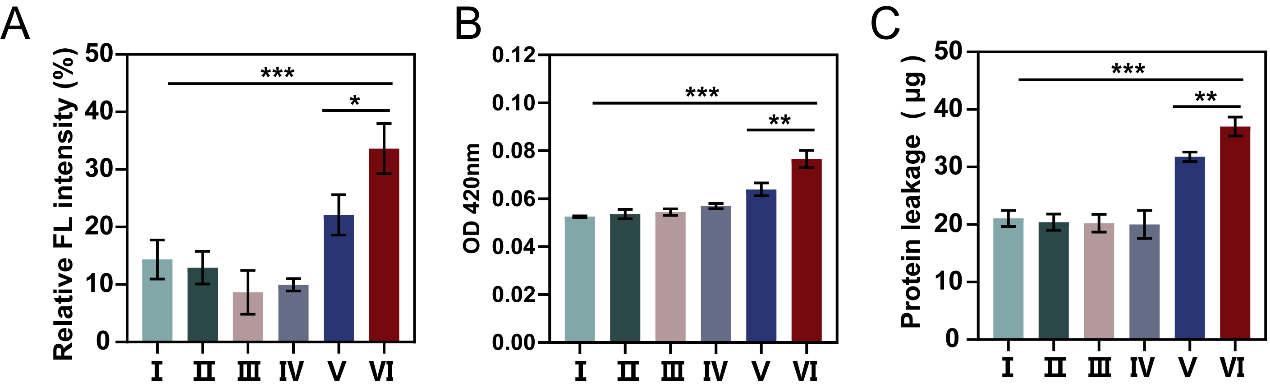
***

**Figure S17.** Proposed mechanism of the antibacterial performance of DR-ZnCuW-LDH nanosheets under US irradiation. (A) *P. aeruginosa* membrane potential after the indicated treatments was monitored with the DiSC_3_(5) probe. (B) *P. aeruginosa* membrane permeability assessed by ONPG hydrolysis assay. (C) Protein release from *P. aeruginosa* quantified using a BCA protein assay kit. PBS (I), PBS +US 5min (Ⅱ), PBS +US 10min (Ⅲ), DR-ZnCuW-LDH nanosheets (Ⅳ), DR-ZnCuW-LDH nanosheets + US 5min (V), and DR-ZnCuW-LDH nanosheets + US 10min (VI). Data are expressed as mean ± s.d. (n = 3), **p* < 0.05, ***p* < 0.01, and ****p* < 0.001.


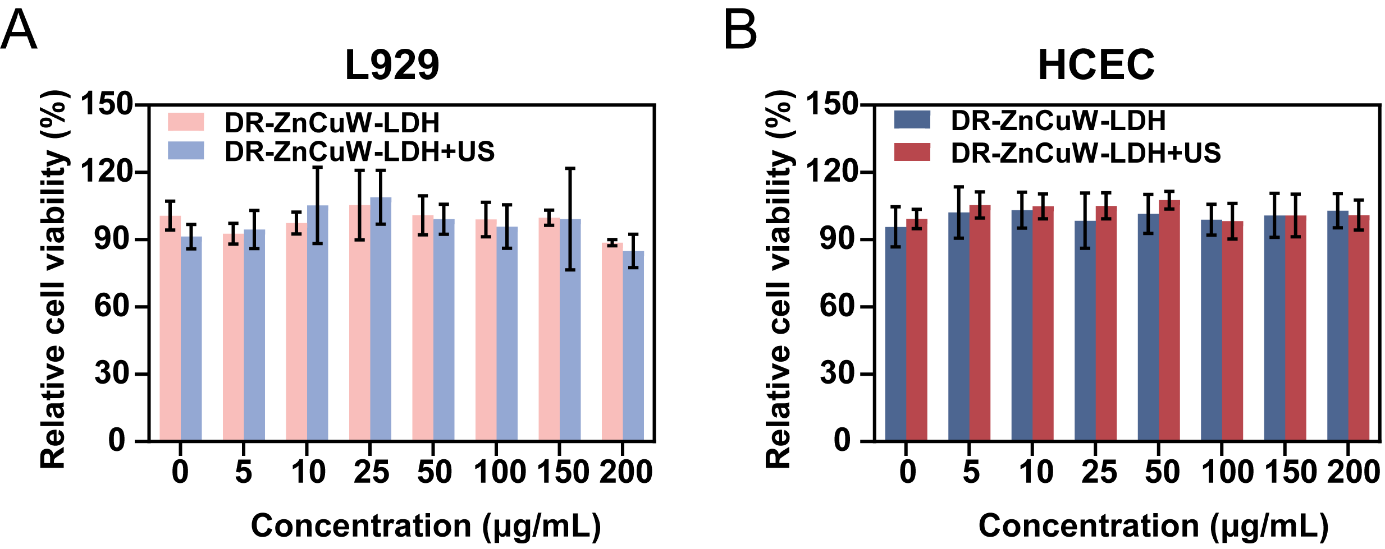


**Figure S18.** Cytotoxicity assessment of ZnCuW-LDH and DR-ZnCuW-LDH nanosheets in *vitro*. (A-B) L929 and HCEC cell lines were treated with different concentrations of ZnCuW-LDH and DR-ZnCuW-LDH with or without US. Data are represented as mean ± s.d. of three independent experiments.


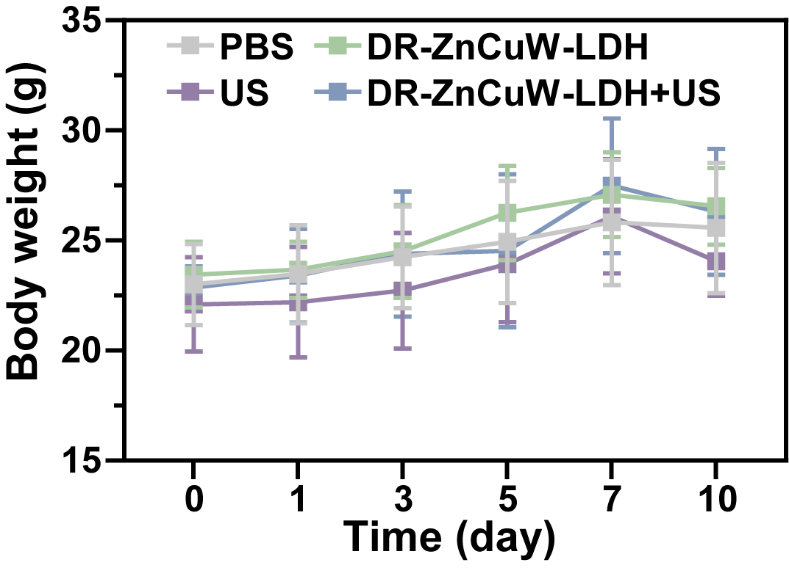


**Figure S19.** The body weight of mice from day 0 to day 10 after the indicated groups. Data are presented as mean ± s.d. (n = 3).


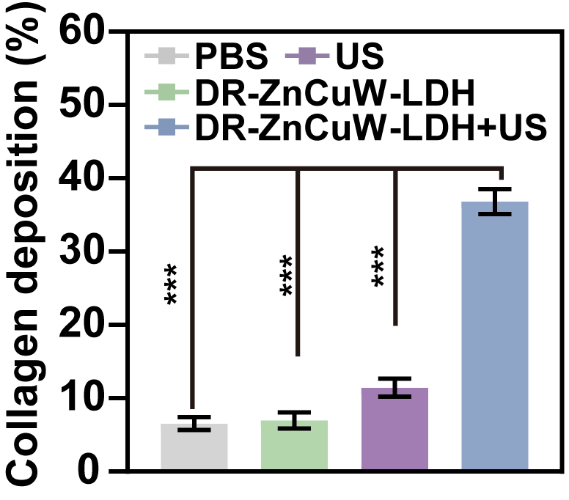


**Figure S20.** Quantification of the relative area of blue-stained collagen deposition percentage of **Figure 4I**. PBS (I), US (Ⅱ), DR-ZnCuW-LDH nanosheets (Ⅲ), DR-ZnCuW-LDH nanosheets + US (Ⅳ). Data are presented as mean ± s.d. (n = 3), **p* < 0.05, ***p* < 0.01, and ****p* < 0.001.


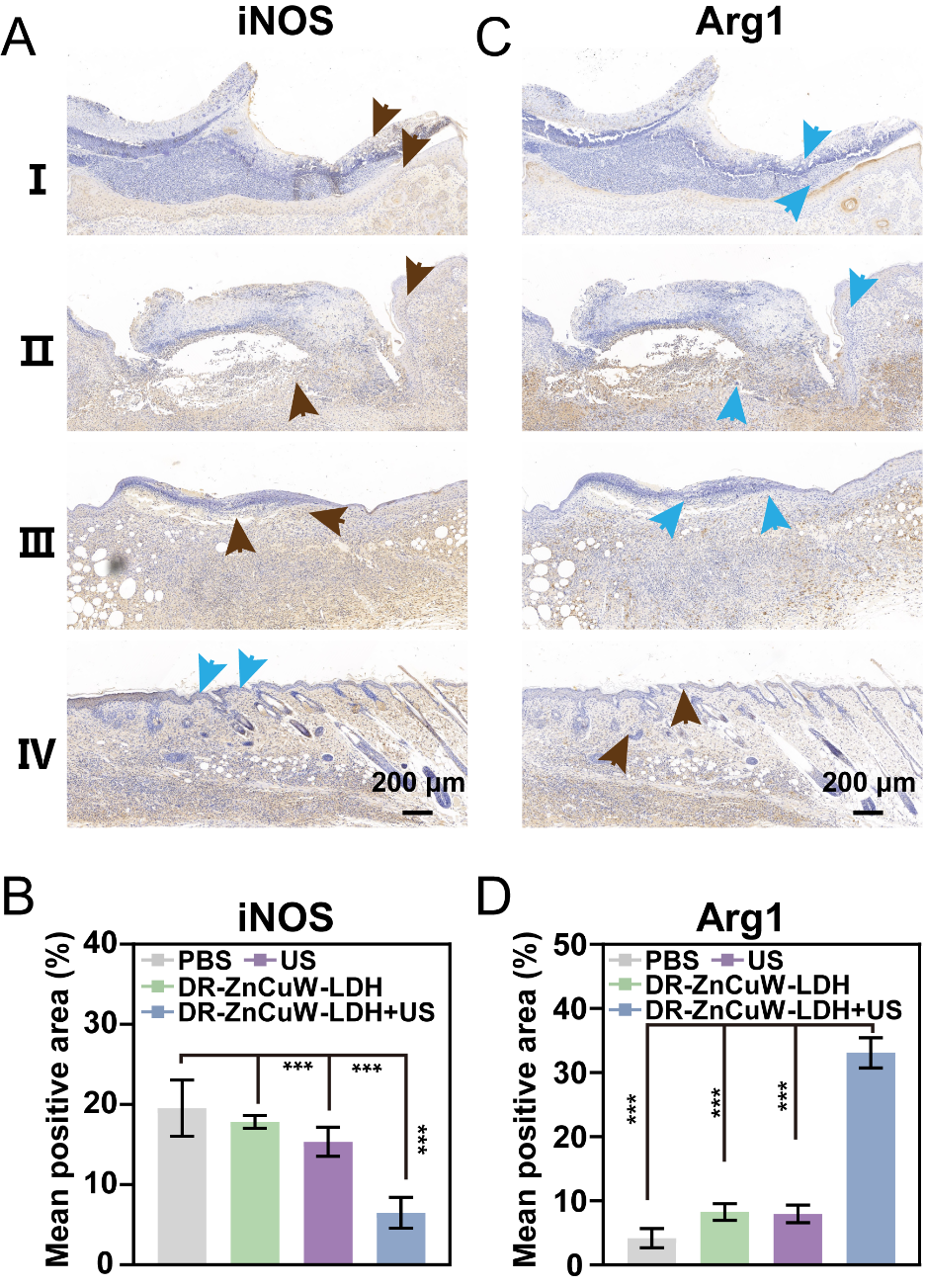


**Figure S21.** Immunohistochemical staining for iNOS (A) and Arg1 (C) in wound sections from different treatment groups. Brown and blue arrowheads highlight iNOS-positive and Arg1-positive regions, respectively. (B, D) Quantification of mean positive area for Arg1 and iNOS, respectively. Data are presented as mean ± s.d. (n = 3), **p* < 0.05, ***p* < 0.01, and ****p* < 0.001.


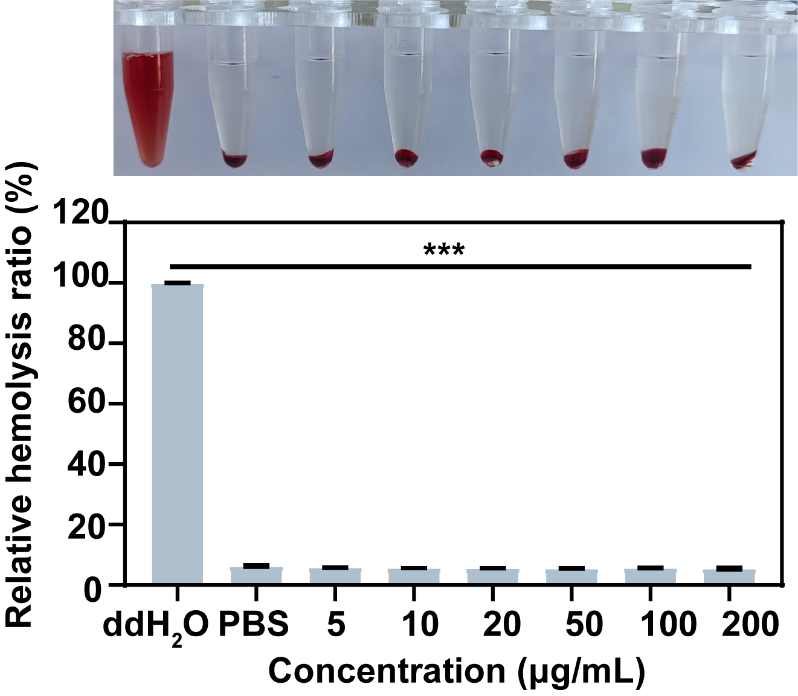


**Figure S22.** Hemolysis ratios of red blood cells incubated with increasing concentrations (5-200 µg mL^-1^) of DR-ZnCuW-LDH nanosheets. Distilled water (ddH_2_O) and PBS served as positive and negative controls, respectively. Data are shown as mean ± s.d., **p* < 0.05, ***p* < 0.01, and ****p* < 0.001.


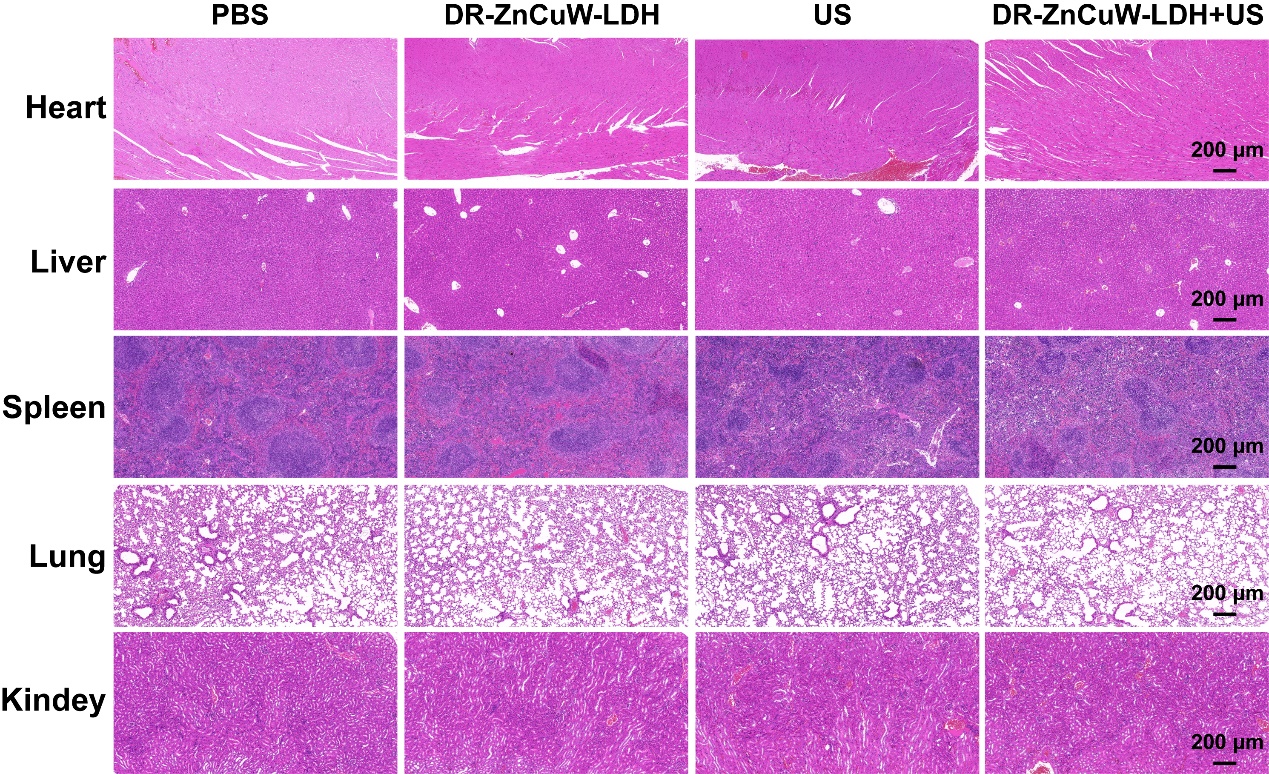


**Figure S23.** Representative H&E of major organs (heart, liver, spleen, lung, and kidney) collected from mice treated with PBS, DR-ZnCuW-LDH, US, or DR-ZnCuW-LDH + US.
